# Metal Nanoparticles‐Enhanced Biosensors: Synthesis, Design and Applications in Fluorescence Enhancement and Surface‐enhanced Raman Scattering

**DOI:** 10.1002/asia.202000847

**Published:** 2020-09-21

**Authors:** Mohammad Tavakkoli Yaraki, Yen Nee Tan

**Affiliations:** ^1^ Department of Chemical and Biomolecular Engineering National University of Singapore 4 Engineering Drive 4 Singapore 117585 Singapore; ^2^ Faculty of Science, Agriculture & Engineering Newcastle University Newcastle Upon Tyne NE1 7RU United Kingdom; ^3^ Newcastle Research & Innovation Institute (NewRIIS) 80 Jurong East Street 21, #05-04 Devan Nair Institute for Employment & Employability Singapore 609607 Singapore

**Keywords:** Metal nanoparticles, Assay design, Plasmonic enhancement, Fluorescence detection, SERS Biosensors

## Abstract

Metal nanoparticles (NP) that exhibit localized surface plasmon resonance play an important role in metal‐enhanced fluorescence (MEF) and surface‐enhanced Raman scattering (SERS). Among the optical biosensors, MEF and SERS stand out to be the most sensitive techniques to detect a wide range of analytes from ions, biomolecules to macromolecules and microorganisms. Particularly, anisotropic metal NPs with strongly enhanced electric field at their sharp corners/edges under a wide range of excitation wavelengths are highly suitable for developing the ultrasensitive plasmon‐enhanced biosensors. In this review, we first highlight the reliable methods for the synthesis of anisotropic gold NPs and silver NPs in high yield, as well as their alloys and composites with good control of size and shape. It is followed by the discussion of different sensing mechanisms and recent advances in the MEF and SERS biosensor designs. This includes the review of surface functionalization, bioconjugation and (directed/self) assembly methods as well as the selection/screening of specific biorecognition elements such as aptamers or antibodies for the highly selective bio‐detection. The right combinations of metal nanoparticles, biorecognition element and assay design will lead to the successful development of MEF and SERS biosensors targeting different analytes both in‐vitro and in‐vivo. Finally, the prospects and challenges of metal‐enhanced biosensors for future nanomedicine in achieving ultrasensitive and fast medical diagnostics, high‐throughput drug discovery as well as effective and reliable theranostic treatment are discussed.

## Graphical abstract

**Plasmonic nanostructures** with anisotropic shapes possess dramatic enhanced electric field when they interact with light. This unique feature has led to the design of metal‐enhanced fluorescence (MEF) biosensors and surface‐enhanced Raman scattering (SERS) biosensors with ultrasensitivity, where metal nanostructure plays an important role as plasmonic enhancer to amplify the optical signals for biological detection.

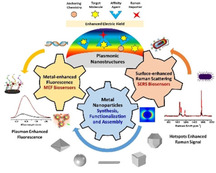

## 1 Introduction

Over the past decades, noble metal nanoparticles have received tremendous attention due to their unique plasmonic properties which can be used to module the optical signal of molecules placing in their vicinity.^1^ For instance, the fluorescence intensity of a fluorophore and Raman signal of a Raman reporter could be enhanced by the plasmonic nanoparticles. These two plasmon‐enhanced phenomena are termed as the metal‐enhanced fluorescence (MEF) and surface‐enhanced Raman scattering (SERS), respectively. Fluorescence and Raman spectroscopy are the two powerful analytical techniques that have been widely used to develop sensitive biosensors for applications ranging from environmental sensing and food safety^2^ to biomedical applications such as early diagnosis and prognosis of diseases.^3^ As the sensitivity of these biosensors relies a lot on their optical signals, further enhancement of the fluorescence and Raman signal intensity via MEF and SERS approaches is a unique advantage to achieve ultralow detection limit even at a single molecule level.^4^

Among the different metal nanostructures, anisotropic gold and silver nanoparticles (NPs) with sharp corners and edges are favourable for the design of MEF‐ and SERS‐based biosensors due to their strong plasmonic enhancement effect under a broad range of incident light wavelengths from the visible to near‐infrared region.^5^ Simulation studies have shown that the anisotropic metal nanoparticles possess dramatically enhanced electric field at their sharp edges and corners when different modes (i. e., electric dipole, magnetic dipole or even higher orders) are being excited under irradiation.5b As the extinction spectra of anisotropic metal NPs shows the size and aspect ratio‐dependency, the extinction spectra of anisotropic metal NPs can be easily tuned to match with the absorbance spectra of the optical sensing probes such as fluorophores and Raman reporters. In particular, the spectral overlap between emission of fluorophores and absorption of metal NPs is the key parameter for designing ultrasensitive MEF biosensors with great enhancement factors.

In this review, we first focus on the synthesis of anisotropic gold and silver nanoparticles and then highlight the most reliable approaches for their shape and size control. For the development of nanoparticle‐based biosensors (also known as nanobiosensors), surface functionalization of metal nanostructures plays very crucial role to enable target specific detection. Thus, different biofunctionalization and assembly methods in preparing the bio‐metal sensing nanoprobes will be discussed. Subsequently, the designs and applications of metal‐enhanced biosensors will be reviewed, which include the recent progress in both planar and colloidal NPs‐based MEF and SERS biosensors. Particularly, the planar‐based MEF and SERS systems were found to be more sensitive than the colloidal biosensors, as aggregation of metal NPs on planar substrate can led to a stronger enhanced electric field in the gap area to form hot spots for detection. Moreover, colloidal biosensors are more suitable for in‐vivo sensing due to the small size of colloidal NPs in aqueous solution similar to most of the biological molecules and cells under physiological conditions. Thus, colloidal biosensors can also be developed into multifunctional probes for theranostic treatment combining diagnostic and therapeutic functions in one application. The success of metal‐enhanced biosensors for future nanomedicine relies on the accomplishment of various technical challenges ranging from theoretical study of metal enhancement effects to standardization of biosensor fabrication methods, as well as further improvement in assay design to achieve single molecule detection for in vivo applications. In addition, development of biocompatible metal‐enhanced biosensors with minimum signal‐to‐noise ratio is of utmost importance for clinical translation, especially for medical diagnostics, drug discovery, and theranostic treatment.

## 2 Synthesis of anisotropic metal nanoparticles

### 2.1 Anisotropic gold (Au) nanoparticles

Gold nanoparticles (AuNPs) of different size and shape could be synthesized under specific reaction conditions.^6^ These AuNPs exhibited unique localised surface plasmon (LSPR) peak depending on their morphology in the visible to near‐infrared regions (above 500 nm wavelength). In general, the LSPR peak experiences a red shift with increasing size of the AuNPs and/or their aspect ratio (i. e., length to diameter ratio). In the following sub‐sections, we review the various synthesis methods leading to the formation of anisotropic AuNPs with good control of size and shape for use in the MEF and SERS sensing systems.

#### 2.1.1 Au nanorods

Gold nanorods (AuNRs) are one of the most popular shapes among the anisotropic AuNPs, due to their well‐established synthesis protocols to control the size, aspect ratio, and monodispersity. Apart from the pioneering works on hard‐templating^7^ and electrochemical^8^ approaches to the formation of AuNRs, the wet‐chemical synthesis of AuNRs with tunable aspect ratios is first reported by Murphy's and El‐Sayed's groups.^9^ Typically, the synthesis AuNRs require the use of small Au seeds (<5 nm), Au^+^ ions (e. g., HAuCl_3_) precursor, cetrimonium bromide (CTAB), trace amount of Ag^+^ ions, and ascorbic acid (AA). While CTAB forms the cylindrical micelle (i. e., >critical concentration for CTAB) to create a nanoreactor for the formation of rod‐shape seed, adding trace amount of Ag^+^ ions to the reaction mixture is important to facilitate the longitudinal growth of AuNRs. In addition, AA allows the slow deposition of Au^+^ ions onto the Au seed, preventing fast nucleation that is unfavourable for the controlled growth of Au nanorods. The as‐synthesized AuNRs normally exhibit the penta‐twinned structure with {111} faces at their two ends and {100} or {110} along the longitudinal faces.9b, 9c, 10 By adjusting the synthesis conditions such as pH and precursor ratios as well as improving the purification methods, researchers are able to enhance the yield of AuNRs (up to 95%) with the aspect ratios of more than 10.9b, 9c, 10 Further studies show that the addition of trace amount of Ag^+^ ions (using AgNO_3_ as the precursor) would enable the high yield synthesis (>95% yield) of single‐crystalline AuNRs with the aspect ratios less than 5.^11^ Besides the seed‐mediated growth approach, some seedless approaches have also been demonstrated by Jana^12^ and Ali et al..^13^ This method is designed based on the in‐situ formation of Au seeds in the growth solution by adding small amount of NaBH_4_ as a strong reducing agent. The aspect ratio can be controlled between 2 and 5 by adjusting the chemical reagents‐to‐gold precursor ratios (i. e., CTAB/AA/NaBH_4_ or CTAB/Au^3+^/Ag^+^) and pH of the reaction mixture. It is worth noting that the synthesis of AuNRs is very sensitive to the impurity present in the precursors. For example, using CTAB obtained from different manufacturer or lot number for the AuNR synthesis might result in variation of particle quality from batch to batch production. It has been shown that the impurity (e. g., iodine in ppm scale) in CTAB resulted in the failure synthesis.^14^

#### 2.1.2 Au nanocubes and other polyhedral nanostructures

**Figure 1:**
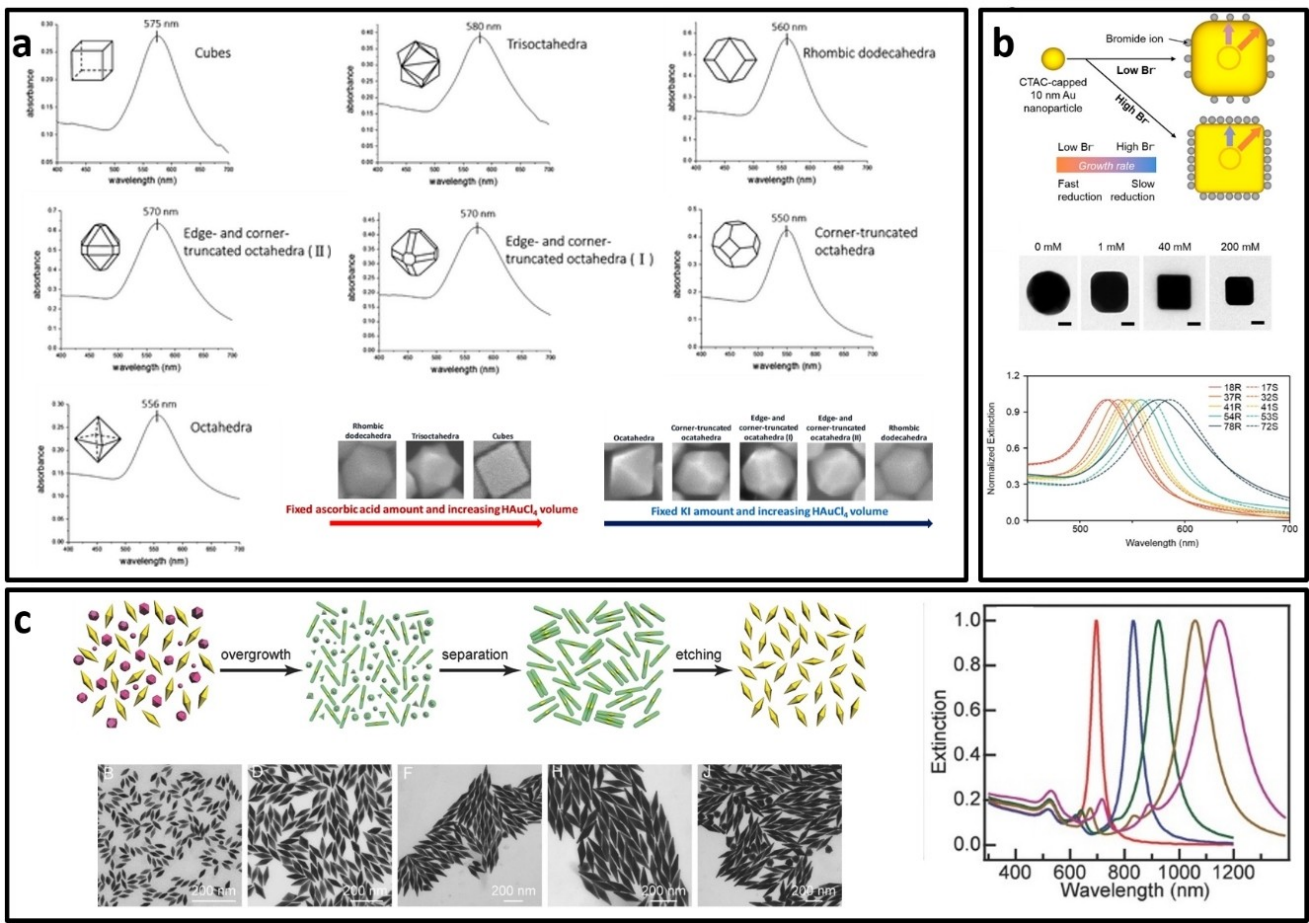
a) Comparison of the extinction spectra of different polyhedral Au nanostructures with average diameter of 90 nm,^23^ b) The role of the amount of Br^−^ in the formation of Au nanocube (top). TEM images of Au nanostructures synthesized at different concentration of Br^−^ ions (middle). The extinction spectra of Au nanocubes with different edge to curvature radius ratio (bottom). R refers to round edges/corners and S refers to sharp edges/corners,^24^ c) Schematic illustration of the purification process of Au bipyramids (AuBPs) by growing silver shell (top left), TEM images of purified AuBPs with different sizes (bottom left) and their corresponding extinction spectra (right).^29^

Synthesis of polyhedral gold nanostructures with different number of facets, edges and corners is of importance due to their wide range of applications from facet‐dependent catalytic properties^15^ to surface‐enhanced Raman scattering.^16^ Various bottom‐up approaches have been developed for the synthesis of Au polyhedral nanostructures, which include (1) using different amount of reducing agent^17^ and gold precursor,^18^ (2) selection of the type of gold precursors (e. g., HAuCl_4_, HAuBr_4_, and AuCl^19^) and shape‐directing ions such as halides (i. e., Br^−^ and I^−^ or Ag^+^),^20^ (3) type of surfactant and its amount,^21^ (4) adjustment of oxidative etching by HCl and (5) tuning the reaction temperature.^22^ Basically, any parameter (i. e., temperature, type of chemicals, and their amounts) that affect the Nernst equation (i.e., free energy of the reaction) could alter the shape of the resulted polyhedral nanostructures. For example, Kuo et al.^23^ have systematically investigated the amount of HAuCl_4_ in affecting the shape of Au polyhedral nanostructures via the seed‐mediated growth approach. Results shows that higher concentration of Au ions led to the formation of Au nanostructures from octahedral to rhombic dodecahedra and cube (Figure 1a).^23^

Among the polyhedral gold nanostructures, Au nanocube is the most well reported nanostructure with tunable size. Park et al.^24^ have successfully synthesized the Au nanocubes of different edge lengths (18–72 nm), which show tunable LSPR peaks from 520 nm to 590 nm. This approach relied on the seed‐mediated growth of the 10 nm cetrimonium chloride (CTAC)‐capped AuNPs in the reaction mixture containing ascorbic acid, CTAC, HAuCl_4_ and sodium bromide. The edge length of the Au nanocubes could be tuned by varying the amount of Au seeds. Bromide ions were found to be favourably adsorbed on the {100} facet,^25^ playing an important role as a shape‐driven agent to form the Au nanocubes. As shown in Figure 1b, a higher amount of Br^−^ ions resulted in the formation of Au nanocubes with sharper edges and corners.

#### 2.1.3 Au bipyramids nanoparticles

Gold bipyramids nanoparticle (AuBPs) are the exceptional nanostructures with promising catalytic and optical properties because of their six {110} facets, which has a higher surface energy than other facets such as {100} and {111} facets,^26^ endowing them with unique catalytic^27^ and optical properties (e. g., electric field enhancement) for various technological applications.^28^ One of the most reliable methods for the synthesis of gold bipyramids was reported by Wang's group using the citrate‐capped Au seeds as the starting reagent, followed by growth of the Au seeds in CTAB growth solution containing specific amount of HAuCl_4_ (0.01 M, 2 mL), AgNO_3_ (0.01 M, 0.4 mL), HCl (1 M, 0.8 mL), and ascorbic acid (0.1 M,0.32 mL) for overnight aging. The sizes of Au bipyramids can be controlled by the amount of added Au seeds.^29^ However, the synthesis of AuBPs usually contains some by‐product (e. g., Au nanosphere).^30^ The same group has thus developed an innovative method to purify the AuBPs through a three‐step process as shown in Figure 1c (top row): i) Growth of Ag on AuBPs and obtaining Au@Ag nanorods/nanowires, ii) Separation of Au@Ag nanorods/nanowires by sedimentation, iii) Etching the silver shell by H_2_O_2_ 29 The as‐synthesized AuBPs have shown higher enhanced electric field compared to Au nanorods. As shown in Figure 1c, the LSPR peak of AuBPs could be tuned from 690 nm to 1155 nm (right) with different length and diameter of the AuBPs (TEM images)

#### 2.1.4 Au nanoplates

**Figure 2:**
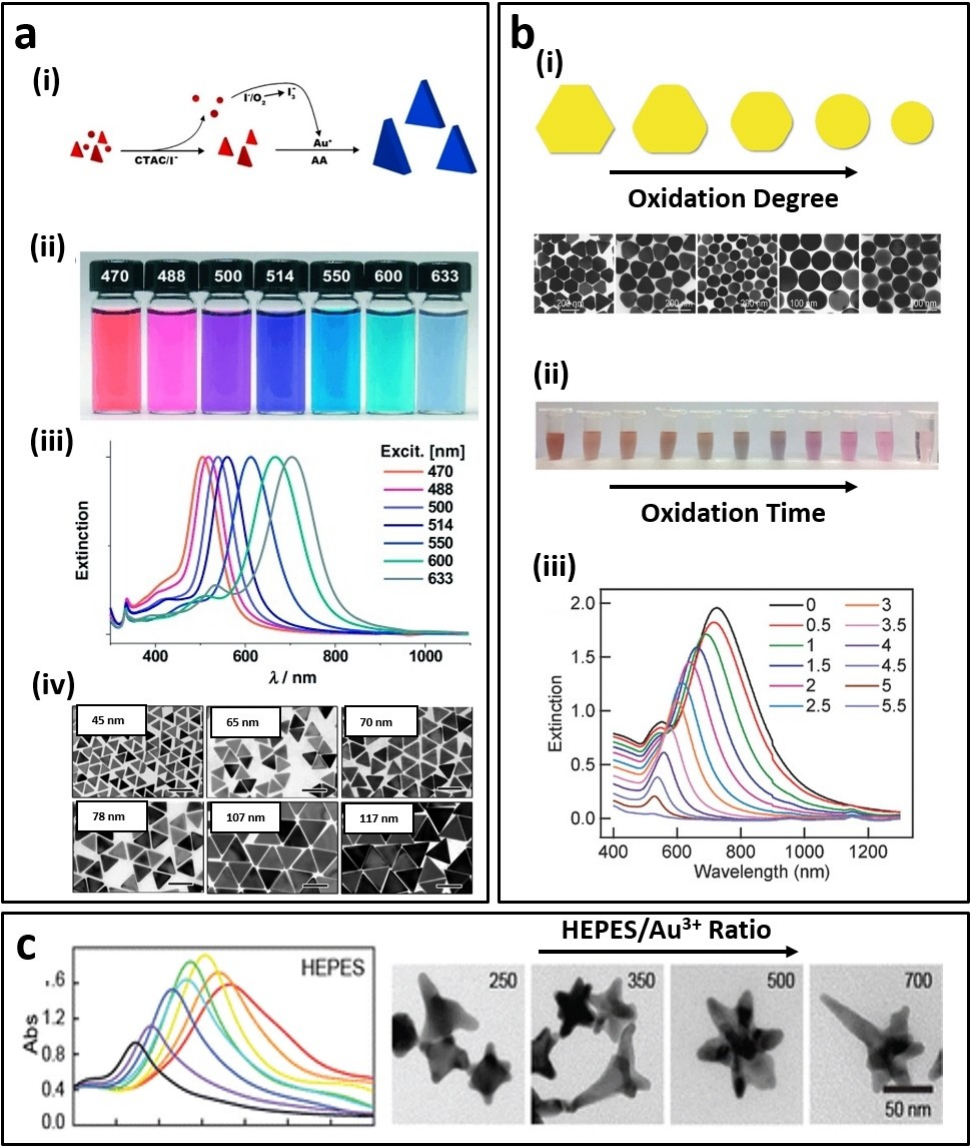
a) Triangular Au nanoplates synthesized by oxidative etching method: (i) Schematic diagram showing the formation mechanism of triangular Au nanoplates, (ii) solution color, (iii) LSPR spectrum and (iv) TEM images of Au nanoplates of different edge lengths.^32^ b) Synthesis of Au nanodiscs using HCl and H_2_O_2_ as etching agents: (i) TEM images and solution color of Au nanoplates at different stages of oxidation process. (ii) The extinction spectra of final product (i. e., spherical nanodiscs) as a function of added H_2_O_2_ volume in mL ^[37]^. c) Synthesis of Au nanostars with multiple sharp tips using different HEPES/Au^3+^ ratio: (left) LSPR spectrum and (right) TEM images of Au nanostars.48a

Au nanoplates are plate‐like nanostructures with two {111} facets exhibiting unique optical properties which can be tuned from visible to NIR region by careful control of their edge length, thickness and morphology (e. g., triangular, hexagonal, disc, etc).^31^ Figure 2a shows the typical extinction spectra of triangular Au nanoplates and their solution colors. In year 2014, Zhang’ group has conducted systematic studies to unravel the role of iodine ions in the synthesis of small Au triangular nanoplates (edge length <100 nm) in high yield.^32^ In this work, a seedless synthesis approach involving the reaction mixture of HAuCl_4_, CTAC, iodine ions and ascorbic acid to synthesize monodispersed triangular Au nanoplates at pH 8 (see the TEM images in Figure 2a). It is proposed that the iodine ions (I^−^) could produce tri‐iodide ions (I^3−^) in the presence of oxygen molecules, where the I^3−^ ions act as the strong chemical etchant to selectively remove the spherical and irregular shapes at an early growth stage under alkaline condition^33^ (Figure 2a). On the other hand, natural molecules such as peptides and aspartic acids have been used to synthesize the anisotropic Au nanoplates without exogenous addition of other chemical agents. In 2010, Tan et al.^34^ reported the first rational design of peptides with tailored‐made sequences and functions to control the growth kinetics and thus the shape and size of Au nanoplates. This biogenic approach is much greener as it uses only the biomolecules as the reducing cum stabilizing agent where the synthesis could be carried out in aqueous solution, neutral pH and room temperature.^35^

Numerous literatures have reported the fabrication of Au nanodiscs using physical methods such as electron‐beam lithography.^36^ The colloidal synthesis of Au nanodiscs was reported by Wang's group.^37^ In this approach, triangular Au nanoplates were first synthesized, followed by the mild oxidation process using HCl and H_2_O_2_ at defined ratio (i. e., HCl: 1 M, 200 μL and H_2_O_2_: 6 wt%, 300 μL) to etch the corners of Au nanoplates. The thickness and diameter of the circular Au nanodisc could be tuned in the range of 10–50 nm and 50–150 nm, respectively. Figure 2b shows the TEM images of Au nanodisc and their solution colour changes during oxidation process. It was observed that the LSPR peak of Au nanodisc red‐shifted to a longer wavelength in the near‐infrared red (NIR) region with increasing aspect ratio (i. e., diameter‐to‐thickness ratio).

On the other hand, star‐shape Au nanoplates can be synthesized using mild reducing agent e. g., ascorbic acid, in the presence of shape–driving agent such as polyvinyl pyrrolydine (PVP)^38^ or H_2_O_2_.^39^ For example, Yamamoto et al. used the ascorbic acid and PVP^38^ for the synthesis of single crystalline star‐shaped nanoplates with six symmetric tips growing in the <112> direction. However, the yield of this method was rather low (∼11%).

#### 2.1.5 Au nanostars

Multi‐branch/spiky gold nanostructures have received tremendous attention due to the strongly enhanced electric field at their tips, enabling red to NIR adsorption with good photothermal effects. Generally, there are two main strategies for the synthesis of gold nanostars, i. e., seed‐mediated growth and seedless approaches, both of which using different chemicals to induce the deposition of Au ions on Au nuclei in different directions.

In seed‐mediated growth approach, the anisotropic growth of Au nanostar is induced by the addition of another metal ions (e. g., Ag^+^ or Fe^2+^) or using surfactants with multiple moieties. This method usually results in the formation of Au nanostars, which contain a spherical core surrounding with multiple tips. The number of tips could be varied from a few to uncountable number of tips (i. e., dense tips).^40^ Practically, the selection of capping agent of Au seeds, type and amount of surfactants,^41^ reducing agent^42^ and added metal ions,^43^ as well as reaction temperature^44^ can affect the synthesis and quality of the final product.^45^

The seedless approach, also called the one‐pot synthesis has been used to direct the formation of Au nanostars by controlling the nucleation rate and growth rate. For example, high yield (90%) of Au nanostars could be obtained in a reaction with citric acid, hydrogen peroxide and bis(p‐sulfonatophenyl) phenylphosphine dihydrate dipotassium as stabilizer.^39^ However, the Au nanostars usually suffer from low stability at room temperature because they tends to transform into the spherical shape which is more thermodynamically stable.^46^ The stability issue of Au nanostars can be addressed by the ligand‐exchange reaction by using the thiol‐capping agents to disperse the Au nanostars in both water and organic solvents.^47^

Another seedless synthesis approach is based on the use of “good buffers” which act as both reducing agent and shape‐driving capping agent for the one‐pot synthesis of Au nanostars. In this approach, the type of buffer molecule, buffer‐to‐Au ions molar ratio and pH of reaction mixtures all play important role in determining the properties of the final product. To date, many studies have reported the successful synthesis of Au nanostars using good buffers, such as 4‐(2‐hydroxyethyl)‐1‐piperazineethanesulfonic acid (HEPES), 3‐(N‐morpholino)propanesulfonic acid (MOPS), and 4‐(2‐hydroxyethyl)‐1piperazinepropanesulfonic acid (EPPS)). Among these “good buffers”, only HEPES could lead to the formation of Au nanostars with asymmetric tips in high yield (>95%)^48^ (Figure 2c). There are several advantages of Au nanostars synthesized by HEPES, which include good particle stability at room temperature, intrinsic biocompatibility, tunable optical properties and high reproducibility with reported extinction coefficients,^49^ making them versatile to be used in different applications such as photonic, sensing, catalysis, etc.

### 2.2 Anisotropic silver (Ag) nanoparticles

#### 2.2.1 Ag nanorods

The synthesis of Ag nanorods was first reported by Murphy's group, where 4 nm citrate‐capped Ag seeds^50^ were used to grow the Ag nanorods in a solution containing CTAB, AgNO_3_ and ascorbic acid, followed by addition of NaOH solution. The sizes of Ag nanorods were tuned by varying the amount of Ag seeds.^51^ However, the reproducibility of this method is rather low due to the difficulty in controlling the reaction conditions such as how to stop the reaction. Recently, polyol synthesis of Ag nanorods has been developed. Typically, ethylene glycol (EG) or short‐length poly ethylene glycol (PEG600) is used as both solvent and mild reducing agent to synthesize Ag nanorods in the presence of PVP as shape‐driven capping agent.^52^ However, this polyol method produced considerable spherical Ag nanoparticles as by‐product.52b In another report, Kitaev et al. employed the 35–45 nm decahedral Ag seeds for the synthesis of Ag nanorods by adjusting the amount of Ag^+^ precursor, reaction time and amount of Ag seeds in the presence of citrate and PVP in water.^53^ While most of the seed‐mediated approaches were based on the use of Ag seeds, Xia's group reported the synthesis of Ag nanorods with tunable length by using 16 nm decahedra Pd nanoparticles as the seeds.^54^ The Pd NPs exist in one of the heads of Ag nanorod while the length of Ag nanorod increases over the reaction time. Apart from the chemical reduction methods, Ag nanorods can be synthesized by photochemical synthesis. For instance, Mirkin's group has reported the synthesis of Ag nanorods with tunable length (330–1010 nm) and diameter (50–60 nm) by adjusting the excitation wavelength of illumination from 600 nm to 750 nm.^55^ Despite the high yield, the Ag nanorods obtained by the photochemical method is considerably large, thus limiting some of their applications.

#### 2.2.2 Ag nanocubes and other polyhedral nanostrcutures

**Figure 3:**
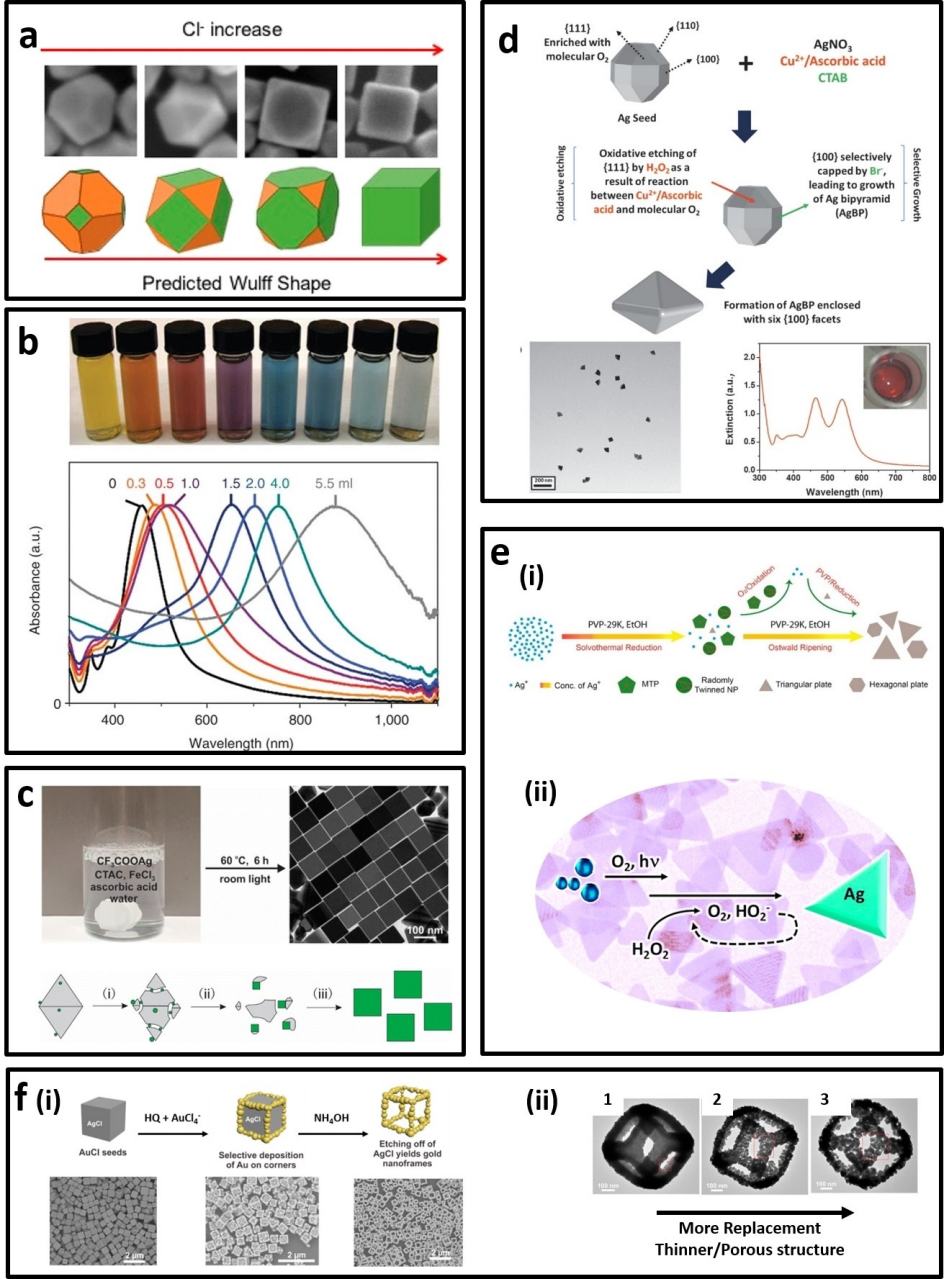
a) Shape evolution of Ag polyhedral nanostructures as function of Cl ions concentration,56c b) Solution color (top) of Ag nanocubes with different edge length synthesized by polyol approach and their corresponding absorbance spectra (bottom). The number on each spectrum refers to the amount of added HCl solution into the reaction mixture .^58^ c) Synthesis (top) and formation mechanism (bottom) of Ag nanocubes with sharp edges/corners using CF_3_COOAg as precursor.59a d) Formation of right silver bipyramids through seed‐mediated growth coupled with in‐situ oxidation approach.5c e) (i) Synthesis of Ag nanoplates in ethanol,^67^ (ii) Photo‐mediated synthesis of triangular Ag nanoplates using H_2_O_2_.^69^ f) (i) Synthesis of AuAg nanoboxes using AgCl as the sacrificial template and etching agent. (ii) TEM images show the formation of thinner/porous AuAg nanoboxes by increasing the galvanic replacement time ^[74]^.

The synthesis of silver‐based polyhedral NPs, particularly Ag nanocubes, relies on the selection of shape‐driving agents to be adsorbed on specific facets of Ag seeds (i. e., nuclei) and guide the direction of growth into Ag polyhedral NPs with well‐defined shape. PVP and halide ions are the most important shape‐driving agents for the synthesis of Ag polyhedral nanostructure.^56^ The synthesis conditions such as temperature, amount of each precursor, type of chemicals and even trace amount of impurity ions directly affect the shape and quality of the final products (Figure 3a). In the case of Ag nanocubes, polyol synthesis in the presence of HCl and PVP has been reported to obtain Ag nanocubes with different edge length as function of reaction time (Figure 3b).^57^ However, this method is highly sensitive to the impurity ions,^58^ where only specific type of ethylene glycol with very low Fe and Cl content could result in high‐quality of Ag nanocubes. While the polyol methods result in truncated Ag nanocubes, Xia's group has reported a new procedure using CF_3_COOAg as the silver precursor, ascorbic acid as the reducing agent, and also Fe^3+^ ions as the shape driving agent to obtain Ag nanocubes with sharp edges (i. e., with average edge length of 35–95 nm) and corners. The growth of cubic structure within the AgCl octahedral formed as a result of mixing CTAC and CF_3_COOAg^59^ (Figure 3c). In another work, the same group reported the synthesis of small Ag nanocubes (<15 nm),^60^ which is not possible to produce by previous methods.^61^

#### 2.2.3 Ag bipyramids

Unlike the gold bipyramids, there are only a few reports on the synthesis of silver bipyramids nanostructures (AgBPs). Typically, the reported procedures for the synthesis of AgBPs requires rather long reaction time (i. e., >8 hr) or involves the use of organic solvent, high temperature, and ultra‐pure chemicals for the high yield synthesis. For example, Wiley et al.^62^ reported the polyol‐approach for the synthesis of Ag bipyramids as results of the formation of twined seeds in the presence of CTAB and ascorbic acid. However, the successful synthesis of AgBPs by this approach usually requires the use of ultrapure polyol (e. g., EG or PEG) with low content of chloride and iron. Another issue is the need of high‐temperature and several washing steps to remove the organic solvent and impurity, raising environmental concerns. In another study, Zhang et al.^63^ reported the synthesis of AgBPs by a photo‐mediated approach with the use of sodium citrate to reduce the Ag^+^ ions in the presence of phenylphosphine dihydrate dipotassium salt (BSPP). However, photo‐mediated approaches require a relatively long irradiation time (*i. e*., >8 hr). More recently, our group have developed a new seed‐mediated approach in aqueous media via the selective growth cum oxidative etching.5c In this method, Cu^2+^ and ascorbic acid react with the oxygen molecules existed on {111} and {110} facets of Ag seeds, resulting in the in‐situ formation of H_2_O_2_ which etches these facets. On the other hand, CTAB capping agent facilitates the growth of {100} facets due to its higher affinity (Br^−^) binding to the {100} facets, which protect them from oxidative etching. The whole reaction takes only 2 hours, which is much shorter than previously reported methods in forming AgBPs (Figure 3d).

#### 2.2.4 Ag nanoplates

Synthesis of silver nanoplates of different morphology like triangular, hexagonal, disc, etc., could be performed via different approaches. Liz‐Marzan's group first reported the PVP assisted synthesis of triangular Ag nanoplates (i. e., with edge length of 60–100 nm) in *N*,*N*‐dimethyl formamide (DMF) as both organic solvent and reducing agent.^64^ However, the yield of this approach was low due to fast reduction at elevated temperature at 150 °C. Later, ultrasound‐assisted Ostwald ripening process was employed by Jiang et al.^65^ to synthesize Ag nanoprism with edge length less than 100 nm. In another attempt, Xia's group employed the slow reduction process in aqueous medium with PVP to reduce Ag^+^ ions. However, large amount of multiple twinned particles (MPTs) was obtained as a by‐product.^66^ Recently, Qin et al.^67^ reported a high‐yield synthesis of PVP‐protected Ag nanoplates in ethanol at 80 °C for 4 hours. They found that the formation of MPTs as final by‐product could be eliminated in this approach due to the complete transfer of Ag atoms from the MPTs to Ag nanoplates *via* O_2_‐mediated Ostwald ripening (Figure 3e, top).

Photo‐mediated aqueous synthesis is also used to obtain silver nanoplates from Ag seeds in the presence of PVP and citrate.^68^ It was found that room light is strong enough to activate the citrate transformation of Ag seeds into Ag nanoplates through preferential binding of citrate ions with the {111} facets of Ag seed. Yu et al. added the hydrogen peroxide to the reaction mixture for the high‐yield synthesis of Ag triangular nanoplates.^69^ They have shown that the presence of light and dissolved oxygen in the reaction mixture play essential roles in the successful synthesis of triangular Ag nanoplates (Figure 3e, bottom).

Besides, Xia's group also reported the synthesis of Ag nanoplates by manipulating the PVP/Ag+ ratio and molecular weight of PVP in the absence of citrate. This study indicated the dual‐functional roles of PVP as both reductant (i. e., due to existence of hydroxyl end group in commercial PVP^70^) and stabilizer in the formation of Ag nanoplates.^71^ Mechanistic mass spectroscopic study revealed that some trimeric clusters (i. e., Ag_3_^+^ or Ag_3_) exist in the nucleation stage of a solution‐phase synthesis using AgNO_3_ as the precursor.^72^ According to Xiong et al.,^73^ thin Ag nanoplates were formed in the presence of polyacrylamide (PAM) due to the formation of its complex with Ag^+^ ions in the solution, slowing down the reduction rate substantially to form the thin plates.

### 2.3 Gold‐Silver (AuAg) bimetallic nanostructures

#### 2.3.1 AuAg Core/shell structures

The synthesis of core/shell nanostructures based on gold and silver usually involves a two‐steps procedure: i) synthesis of core and ii) growth of shell. The important parameters of the synthesis include mild reducing condition (e. g., using light) for growing the shell and adjusting the ratio of the metal core (as seeds) to metal ions (as precursor for shell growth).^75^ The AuAg or AgAu core/shell nanoparticles could be synthesized using different shapes of metal nanoparticles as core, while the final shape of resulted nanostructure depends on the synthesis condition of shell growth.^76^

#### 2.3.2 AuAg alloys

##### 2.3.2.1 Co‐reduction method

Co‐reduction method have been mostly used to develop spherical AuAg alloy nanoparticles,^77^ while some literature were found to apply this method for synthesis of other nanostructures such as triangular nanoplate,^78^ nanorod,^79^ nanowire,^80^ etc. In this approach, the reduction of Ag^+^ ions and Au^3+^ ions usually occur simultaneously. By varying the ratio between the two metal precursors, the compositions and thus the optical properties of the final AuAg alloy nanoparticles could be tuned between the absorption wavelength of the pure AgNPs and AuNPs in the visible region.

##### 2.3.2.2 Galvanic replacement

Galvanic replacement method relies on the difference between the reduction potential of gold (Au^3+^+3e^−^→Au, E=+1.498 V) and silver (Ag^+^+e^−^→Ag, E=+0.7996 V).^81^ This potential difference leads to the replacement of Ag atoms by Au atoms in a mixture of Ag nanoparticles and Au precursor ions (i. e., HAuCl_4_). Through the careful control of the reaction time, this method can lead to formation of alloy AgAu nanostructures, which are mostly hollow.^82^

##### 2.3.2.3 Bimetallic nanoframes and nanoboxes

Nanoboxes have received tremendous attention due to their unique catalytic and optical properties.^83^ Gold nanoframes are hollow nanostructures that exhibit LSPR peak in NIR region, making them highly suitable for photothermal therapy and surface‐enhanced Raman scattering using NIR laser wavelength. There are two general approaches for the synthesis of Au nanoframes. The first approach is based on the use of a sacrificial template such as AgCl for the growth/deposition of gold atoms on it, followed by removing the template using etchant such as ammonium hydroxide (NH_4_OH) (Figure 3f (i)).^74^ The second approach is based on the galvanic replacement of Ag atoms by Au atoms. The synthesis of Au nanoframes using this approach usually starts from synthesis of a silver nanostructure (i. e., nanocube or other polyhedral nanostructures) followed by purification and addition of Au precursor for galvanic replacement reaction. It was found that more replacement of Ag atoms by Au atoms results in thinner frames with higher porosity (Figure 3f (ii))^74^ and tunable optical properties.^84^ Interestingly, Au nanoframes of different morphology can be obtained by using different Ag nanostructures as the starting seeds.^85^ The synthesis of Au nanoframes is not limited to the use of Ag nanocube as seed, the plate‐like nanostructures such as Ag nanodisc and triangular nanoplate have also been used for the synthesis of Au nanoframes (i. e., circular and triangular nanorings). Similar approach have been used to synthesize other bimetallic nanostructures with good porosity and stability such as monometallic Pt hollow nanoboxes using Ag*‐Pt core‐shell nanocubes as sacrificial templates as reported by Tan et al..^83^

## 3 Functionalization and Assembly of Hybrid Metal Nanostructures

Capping agents are usually used in the aqueous synthesis of anisotropic metal nanoparticles to stabilize and direct the growth of anisotropic metal nanoparticles. In some cases, the capping molecules (i. e., citrate, tannic acid, CTAB, etc.) are loosely bound to the surface of metal NPs which could interact with other charged molecules *via* electrostatic interaction or easily replaced by molecules of higher affinity such as thiol molecules or polymers for higher particle stability. In addition, surface functionalization allows the direct or self‐assembly of bio‐metal nanostructure suitable for sensing applications. Particularly, biofunctionalization of metal nanoparticles is out most important to exert biorecognition (e. g., antibody‐antigen binding) to detect specific target biomolecule. The biomolecular interaction can also be used to assemble the optical probes (e. g., fluorophores) and metal NPs to form the sensing probes for MEF and SERS. In the following session, we will discuss the different strategies to functionalize the surface of metal nanoparticles for metal‐enhanced biosensors.

### 3.1 Biorecognition‐induced assembly

**Figure 4:**
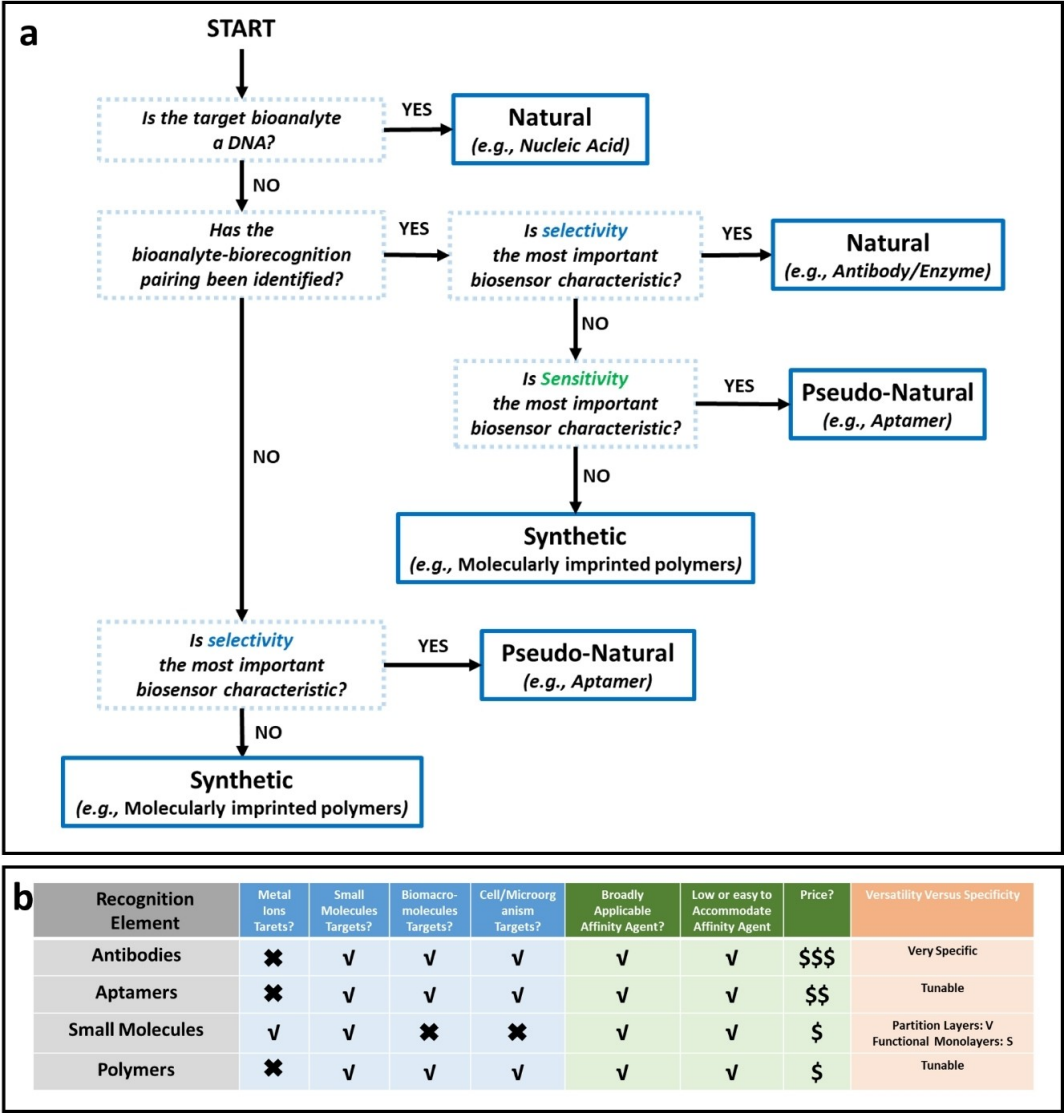
a) Decision map to select the right biorecognition element for biosensor designs ^[91]^, b) A comparison of different recognition elements including antibodies, aptamers, small molecules and polymers.^93^

Biofunctionalization, which enable biological recognition on metal nanoparticles, is one of the most important aspects in designing metal‐enhanced biosensors. Affinity binding in the biological systems often acts as the “lock and key”, where specific pair of biomolecules such as antibody‐antigen,^86^ protein‐DNA^87^ and protein‐peptide,^88^ as well as artificially selected aptamer‐target molecule (ranging from metal ions, small molecules and proteins to more complex targets such as whole cell)^89^ could interact with one another specifically, leading to the design of highly selective biosensors. In addition to these biomolecule pairs, synthetic molecular‐imprinted polymers have been developed to interact with specific molecule of interest.^90^ The key factor in biosensors design largely depends on the selection of biorecognition element. Morales and Halpern^91^ have reviewed different types of biorecognition elements and drawn a decision map that can be used to select a recognition element in the initial design of biosensors (Figure 4a). The sensitivity of a biosensor also depends on the number of available binding sites per surface area, equilibrium dissociation constant, and steric hindrances. The density of available sites is in relationship with the size of biorecognition element, where aptamers and DNAs (∼1–2 nm in size) result in higher surface coverage as compared to antibodies (∼10–15 nm in size).86a In addition, steric hindrances also affect the conformational change in the biorecognition element attached to the surface (i. e., especially antibodies), resulting in inaccessibility of the binding site and therefore, reduces the sensitivity of the biosensors.^92^ Figure 4b shows the comparison of the binding affinity, possible target analytes and prices of different biorecognition agents.^93^

These biorecognition elements are not only important for target‐specific binding, but also useful in particle stabilization and to direct the assembly of nanoparticles for biosensor development. For example, our groups have developed several colorimetric and DLS ‐based nanoparticle biosensors using different biorecognition‐induced aggregation mechanisms to detect various analytes from small drug molecules, DNA/RNA, protein biomarkers to cancer cells.^94^ SERS is another sensing system often employ the biorecognition to induce aggregation of metal NPs in forming the hotspots to enhance its detection signal. Some target molecules such as protein may interact with more than one active sites on the surface of different particles, leading to the aggregation of metal NPs and thus enhanced electric field. This strategy has been used to improve the sensitivity of MEF and/or SERS biosensors.

### 3.2 DNA directed assembly

**Figure 5:**
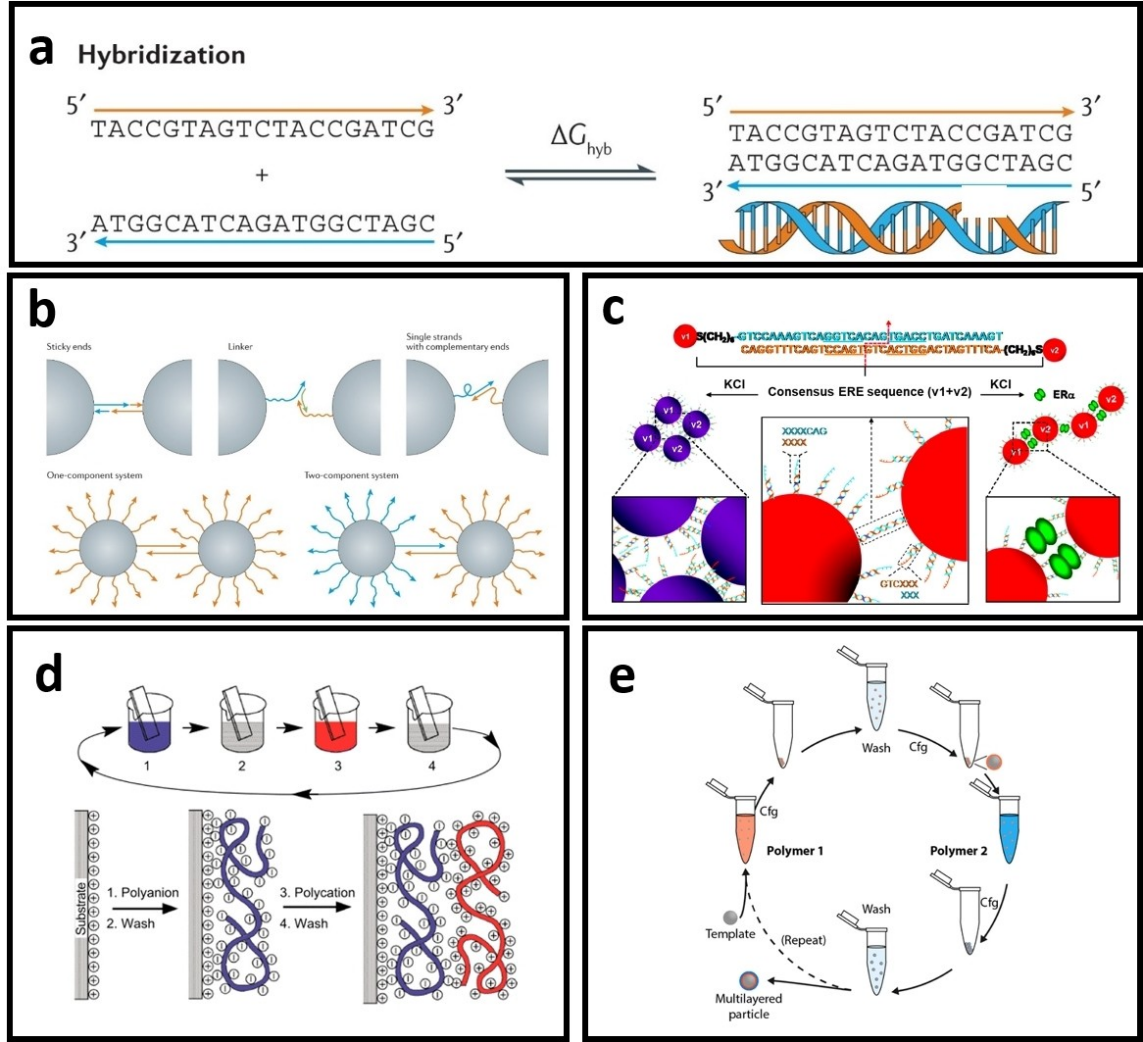
a) Hybridization of two complementary single‐stranded DNAs, b) Bridge formation between the two DNA‐grafted particles with sticky ends or complementary sequences,^96^ c) dsDNA with short complementary stick ends to form the segmented dsDNA‐modified AuNPs for colorimetric biosensor development,95a d) Deep‐coating approach for the layer‐by‐layer (LBL) assembly of differently charged polyelectrolytes on the planar substrate,^102^ e) Steps in the LBL assembly of the oppositely charged polyelectrolytes in forming the multi‐layered particle.^103^

Among the aforementioned biorecognitions, DNA hybridizations is often employed to direct the metal nanoparticle assembly for the plasmonic biosensors design based on the aggregation‐induced solution color (i. e., colorimetric biosensor) or size changes (i. e., DLS nanosensor).94c, 94d, 94e, 95 The structure of a single‐stranded DNA (ssDNA) consists of a linear combination of four nucleotides: cytosine (C), adenine (A), guanine (G), and thiamine (T). Complementary ssDNAs will hybridize through base pairing (i. e., G binds with C and T binds with A) to form the double‐stranded DNA (dsDNA) with double‐helical structures (Figure 5a). Typically, thiolated DNAs is used to functionalize the metal NPs94c, 94d, 94e, 95a, 95b and form the DNA‐hybridized metal nanostructures via: i) ssDNA‐NPs with complimentary sequences ii) ssDNA‐NPs with complementary sticky ends and ii) target linker hybridize with two sets of ssDNA‐NPs (Figure 5b) as well as the (iv) dsDNA‐NPs with short complementary dangling ends (Figure 5c).95a, 95b, 96 For metal‐enhanced biosensors such as MEF and SERS, the length of DNA is critical to control the distance between sensing elements (i. e., fluorophores or Raman reporter) and metal NPs. For instance, the distance between the fluorophore and metal NP in MEF will need to be optimized to prevent fluorescent quenching, while a short distance between the metal NPs and Raman reporter (i. e., <3 nm) is desired for SERS applications. Furthermore, the length and sequences of DNA can be varied by the number of nucleotides from a few angstroms to more than 10 nm. This is a unique advantage of using the programmable biomaterials (e. g., DNA) to precisely assemble metal NPs with biorecognitions for biosensors development.^96^, ^97^

### 3.3 Layer‐by‐layer assembly

Besides biorecognition, non‐specific interactions such as electrostatic attractions can be exploited for nanoparticle assembly. Particularly, layer‐by‐layer assembly (LBL) technique has been widely used to build up layers of polyelectrolytes on either planar surfaces or surface of metal nanoparticles (NPs). This method uses electrostatic interactions between differently charged polyelectrolytes to assemble different number of bilayers on the substrate or NP surfaces. The self‐assembly process of each pair of polyelectrolyte bilayers can be repeated until reaching the desired thickness on surfaces. LBL assembly can be employed to control the distance between metal nanoparticle and fluorophore in the MEF biosensor.^98^ In 2020, we investigated the distance‐dependent MEF effect of a newly synthesized aggregation‐induced emission photosensitizers (AIE‐PSs) with silver nanoparticles (AgNPs) using LBL assembly method. Briefly, polyethylenimine and poly (sodium 4‐styrenesulfonate) are used to assemble AgNPs in forming the Ag@AIE‐PS for up to three bilayers, achieving a 6 times fluorescence enhancement in the AIE‐PS.^99^ In addition, biomolecular interactions such as biotin‐avidin, antibody‐antigens, complementary ssDNAs, and lectin‐carbohydrate can be used to form the bilayers with controlled thickness in the nanometre range for various applications.^100^ For example, Tan et al.35a have reported the use of LBL‐assembled polypeptide multilayer films as reactive templates for in‐situ morphosynthesis of small gold nanoplates. The selection of polyelectrolytes for LBL as well as their charge density and molecular weight, pH, temperature, and ionic strength of the solutions are the key factors in determining the thickness of multilayer films.^98^, ^101^ Despite that LBL is very easy to perform on the planar system (e. g., glass slides or other surfaces) by deep coating and washing steps (Figure 5d),^102^ it is relatively challenging for the colloidal nanosystems as the separation step of nanoparticles by centrifugation may lead unfavourable NPs agglomeration (see Figure 5e),^103^ where some of the NPs may lose in each washing/centrifugation step resulting in low yield production of multi‐layered colloidal NPs.^104^

## 4 Biosensor designs based on metal‐enhanced fluorescence (MEF)

### 4.1 Principles of MEF

**Figure 6:**
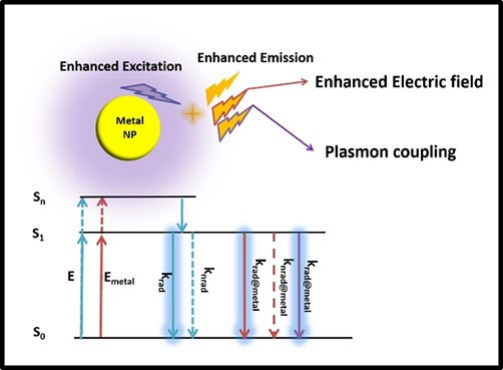
Jablonski diagram illustrating the principles of metal‐enhanced fluorescence for a classical fluorophore.

When a classical fluorophore is excited under specific excitation wavelength, its electrons go from the ground state (S_0_) to the excited singlet state (S_1_). As illustrated in the Jablonski diagram in Figure 6, the excited electrons will lose their energy via two different ways, i. e., non‐radiative decay and radiative decay, before returning to the ground state. The excitation rate is proportional to the square of electric field caused by the interaction of excitation light and environment. Once a fluorophore is placed in the vicinity of a metal NP, the excitation rate, non‐radiative decay rate and radiative decay rate of the fluorophore will change. The excitation rate is enhanced due to the enhanced electric field around the metal NPs. The enhanced excitation rate results in a higher population of electrons in the excited state, and therefore, the rate of radiative and non‐radiative decays will increase. The enhanced electric field reaches its maximum at the surface of metal NPs and decreases exponentially to the far distances. In addition, energy transfer from the excited fluorophore to metal NP is another non‐radiative process, which reduces exponentially with increasing distance between the fluorophore and nanoparticles. Due to the competition between the enhanced non‐radiative decay rates and radiative decay rates, the fluorescence usually quenched for a very short separation distance (<5 nm), resulting in dramatic decrease in quantum yield. Depending on the optical properties of a particular fluorophore (i. e., excitation, wavelength, emission wavelength and quantum yield) as well as the size, shape, and composition of metal NPs, fluorescence intensity reaches a maximum at an optimum separation distance (i. e., ranging from 5 nm to 30 nm in most of the reported MEF systems). The fluorescence intensity normally decreases for a separation distance larger than the optimum value until reaching its original intensity of fluorophore at a very far distance. This distance‐dependent phenomenon is called the metal‐enhanced fluorescence (MEF) which was first observed by Geddes and Lakowicz in 2002.^105^ In short, fluorescence enhancement is a function of the enhanced electric field (which decreases exponentially) and enhanced quantum yield (which increases exponentially) with the separation distance between fluorophores and metal NPs.

As MEF is a distance‐dependent phenomenon, precise control of the separation distance between metal nanoparticle and fluorophore (or gap) is an important issue in designing biosensor. This gap is usually made by a dielectric material. The dielectric layer could be silica, polymer or even biomolecules that have been discussed extensively in the literature.^106^ The biorecognition element can be attached to this dielectric layer or the surface of metal NPs to create optimum distance (*i. e*., usually>5 nm) between the fluorophore and metal NPs to achieve the maximum fluorescence enhancement factor.

### 4.2 Type of MEF biosensors

There are two types of MEF biosensors based on the use of metal nanostructures in either planar or colloidal systems. Table 1 summarizes the recent advancement of using MEF sensing principles for biosensor designs. As can be seen, various MEF biosensors have been developed to detect a wide range of analytes such as metal ions, ADP/ATP, virus antigen, etc. using different types of metal nanoparticles and fluorophores.

**Table 1:** Recent advances in designing colloidal MEF biosensor systems.

Metal NPs/Size	Dielectric/Thickness	Colloidal Or Planar	Fluorophore	Analyte (Medium)	Linear range & Limit of Detection	Biosensor Mode	Ref
Au nanocube 50 nm	Silica 21 nm	Colloidal	5‐carboxyfluorescein	Pyrophosphate (Solution and In‐Vitro)	2–50 pM/1.3 pM	Turn On	107
Ag nanoparticle 40 nm	Silica 7 nm	Colloidal	2‐aminoanthracene	2‐aminoanthracene (solution)	1–800 nM/1 nM	Turn On	108
Ag nanoparticle	Silica 10 nm	Colloidal	Au nanoclusters	Cu^2+^ ion (Solution and In‐Vitro) inorganic pyrophosphate pyrophosphatase (Solution and In‐Vitro)	0.05–0.8 M/39 nM 0.5–60 μM/78.7 nM 5–100 mU/0.976 mU,	Turn off Turn on Turn Off	109
Ag nanoparticle 30‐50 nm	Silica 30 nm	Colloidal	FITC‐anti IgG	anti IgG (Solution)	–/1.5 ng/mL	Turn On	110
AuNS@Agnanocube 10 nm@13.5 nm	Direct	Colloidal	Rhodamine	Hg^2+^ ion (Solution)	0.001–1000 ppm/0.94 ppb	Turn On	111
Au nanorod 44 nm×19 nm	Silica 22 nm	Colloidal	TCCP dye	Na_2_S (Solution) H_2_S (In‐vitro)	0.039–12.5 μM/17 nM	Turn On	112
Au nanorod 83 nm×25 nm	Polystyrenesulfonate/A monolayer	Colloidal	Rhodamine 6G	Cysteine (Solution)	0.01 to 2.5 μg/mL	Turn On	113
Ag nanoparticle 50 nm	Silica 30 nm	Colloidal	Europium (Eu3+)‐ tetracycline complex	Tetracycline (Solution)	0–6 μM/ 83.1 nM	Turn On	114
Au nanoparticle 6.55 nm	Complementary ssDNA	Colloidal	Alexa fluor 488 dye	single‐stranded nucleic acids (Solution)	0.1–7.5 nM/372 pM	Turn On	115
Au bipyramid 751 nm LSPR peak	Aptamer	Colloidal	Cy7	adenosine triphosphate	0.2–10 μM/35 nM	Turn On	116
Ag nanoparticle 50 nm	Silica (8 nm) and dsDNA	Colloidal	PicoGreen	adenosine triphosphate (Solution)	100 nM–10 mM/14.2 nM	Turn Off	117
Magnetic Au nanorod 745 nm×267 nm	molecular beacon‐25 (25 bps)/8.5 nm	Colloidal	5(6)‐carboxyfluorescein	Exosomal miRNA‐124 (Solution and In‐Vitro)	10^−12^‐10^−6^ M/‐	Turn On	118
Au nanoparticle 10–15 nm	Cysteamine‐glutaraldehyde	Colloidal	Thionine	Hepatitis B Virus Surface Antigen (Solution)	4.6×10–9–0.012 ng/mL/4.6×10‐9 ng/mL	Turn On	119
Au nanorod 49 nm×18.5 nm	Silica 16 nm	Colloidal	Organic fluorescent probe	γ‐glutamyl transpeptidase In HEP‐G2 cell (Solution and In‐Vitro)	0–1 U/L/1.2 mU/L	Turn On	120
Au NP/–	Oligonucleotide	Colloidal	FAM dye	Escherichia coli O157 : H7 Salmonella serotype Choleraesuis Listeria monocytogenes	37–3.7×10^7^ CFU/mL/34 CFU/mL 30–3×10^7^ CFU/mL/6.4 CFU/mL 32–3.2×10^7^ CFU/mL/70 CFU/mL	Turn On	121
Au nanoparticle 20 nm	Fumed Silica particle 5 μm	Colloidal	CdSe@ZnS quantum dots	Formaldehyde (Solution)	0.5–2.0 ppm/–	Turn Off	122
Ag nanoisland (Commercial Silvered 96‐well plates, or Quanta Plates™)	YeBF protein	Planar (96‐well microplate)	Fluorescein sodium salt (FITC)	Trypsin (Solution)	10‐1×10^5^/1.89 ng	Turn off	123
Ag nanoparticle/30–50 nm	Silica/15.5 nm	Planar	FITC	Anti IgG (Solution)	–/1.5 ng/mL	Turn On	110
Ag nanoparticle/ 68 nm	Rabbit IgG‐Goat Anti‐Rabbit IgG	Planar	Cy5	Goat Anti‐Rabbit IgG (Solution)	–/–	Turn On	124
Au nanoparticle 100 nm	Silica 10 nm	Planar	Nile Blue	Immunoglobulin‐M	10–500 ng/mL/5 ng/mL	Turn On	125
Au bipyramid 58 nm×22 nm	Biotin‐streptavidin	Planar (paper)	Alexa 680	Biotin or Streptavidin	–	Turn On	126
Au nanoparticle 20 nm	Anti‐mouse‐ Anti‐Megalin Anti‐mouse‐ Anti‐Podocin	Colloidal	Alexa Fluor 488 Alexa Fluor 647	Megalin in the tubules (In–Vivo) podocin in the glomeruli (In–Vivo)	–	Imaging	127
Au nanoparticle 14–20 nm	–	Colloidal	thiophene based Schiff N,N’‐bis(thiophene‐ 2‐ylmethylene)thiophenemethane Nanoparticle (100–180 nm)	cysteine (In‐Vitro) cytosine (In‐Vitro)	–	Imaging	128
Ag nanoparticle 30 nm	Dopamine‐Formate complex	Colloidal (AIE)	Tb3+‐Dopamine‐formate complex	Dopamine (Solution)	0.5–100 nM/0.15 nM	Turn On	129
Au@Ag nanoparticle 30 nm	Oligonucleotide 8.2 nm	Colloidal (AIE)	Cy5	DNA (Solution)	–/3.1 pM	Turn On	130
Au@Ag nanoparticle 19 nm @5.6 nm	Cysteine	Colloidal (AIE)	rhodamine B isothiocyanate	Cysteine	0–34 pM/3.4 pM	Turn On	131

#### 4.2.1 MEF biosensors designs using planar substrate

One of the most popular methods for developing MEF biosensors is the deposition of pre‐synthesized metal nanoparticles on the surface of a planar substrate through either electrostatic interaction or chemical bond. The planar substrates include hard‐substrate (i. e., glass slide, Si wafer, etc.) and flexible materials (i. e., paper, PDMS, flexible polymers). As mentioned previously, the distance between the metal NPs and fluorophore plays an important role to the fluorescence enhancement. Typically, this distance can be controlled by the recognition elements themselves (i. e., DNA, antibody, aptamer, etc.) or through the addition of a secondary dielectric layer on the metal NPs surfaces before conjugation with the biorecognition element. Depending on the biosensor design, it is also possible that the analyte molecule itself act as the fluorophore in the MEF system.

**Figure 7:**
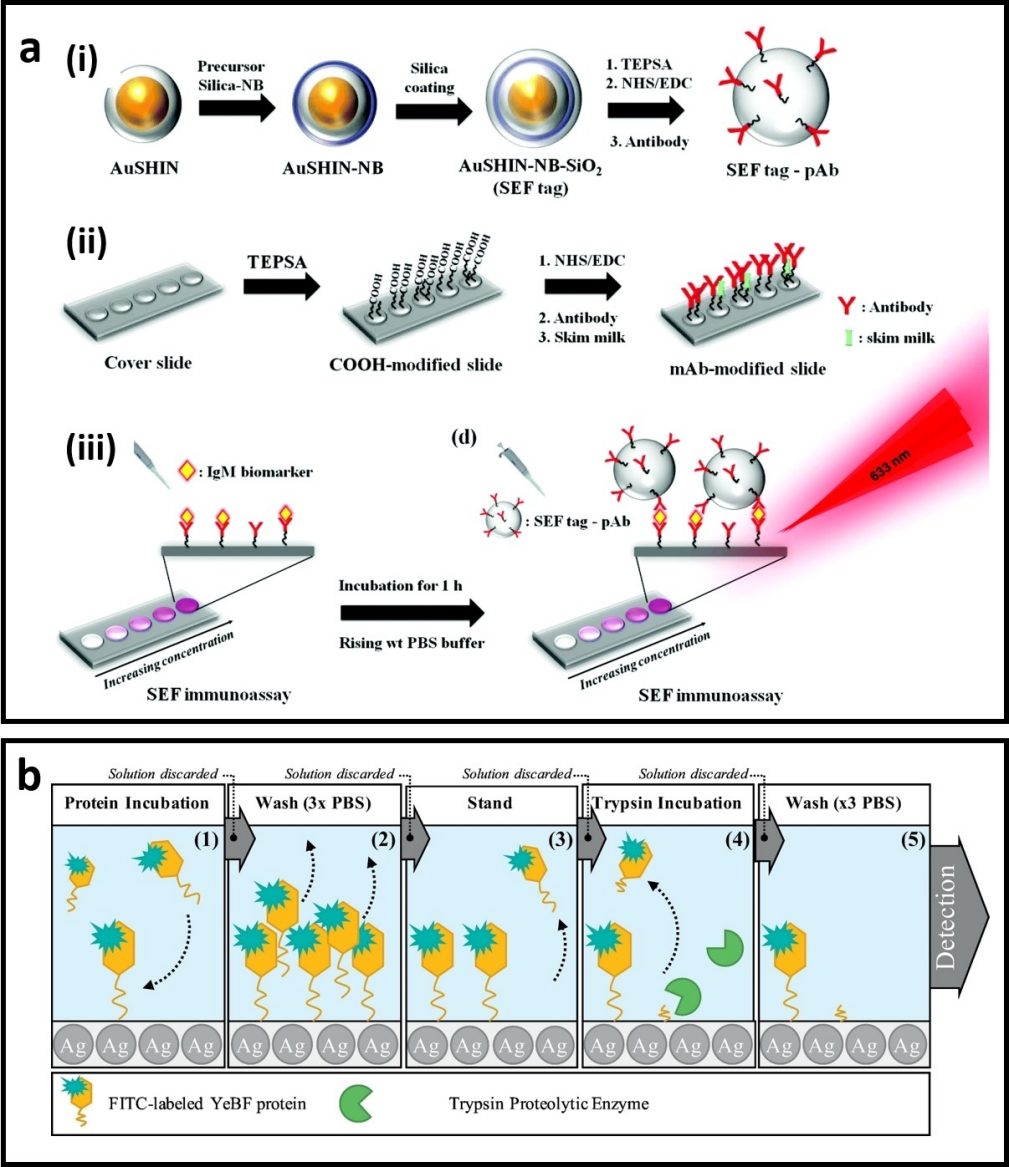
a) Schematic illustration of (i) surface‐enhanced fluorescence (SEF) tag ‐pAb preparation using shell‐isolated gold nanoparticle with a layer of Nile blue and another layer of silica followed by conjugation with antibody, (ii) Surface functionalization of glass slide by (3‐triethoxysilyl) propylsuccinic anhydride (TEPSA) to obtain carboxylic group on the surface, followed by antibody conjugation, (iii) Sandwich‐like structure for detection of Immunoglobulin‐M in the milk through metal‐enhanced fluorescence,^125^ b) Schematic illustration of the experimental procedure and assay construction for ultrasensitive trypsin detection using silvered 96 well plates.^123^

Metal‐enhanced fluorescence has been extensively studied using glass slides as the substrate. The glass slide should be cleaned by either aqua regia solution or light treatment (UV light and UV ozone) or plasma cleaner. This cleaning procedure is essential to remove any organic substance from the surface and make it more hydrophilic with surface charges for subsequent modification. It is easy to deposit the metal NPs onto the glass surface through electrostatic interactions or silane chemistry to form the chemical bond. After which, the metal NP‐deposited glass slide can be functionalized with the biorecognition elements (e. g., antibody, aptamer, etc.) to develop the sandwich structure for biological detection. Camacho et al.^125^ have developed the biofunctionalized Au@Silica core/shell nanoparticles (i. e., 100 nm Au core and 10 nm silica shell) to detect the Immunoglobulin‐M. As shown in Figure 7a, the Au@Silica‐Nile Blue@Silica nanoparticles were formed by a thin layer of silica on Nile blue fluorophore‐loaded Au@silica NP followed by the functionalization of polyclonal IgM antibody. This unique sandwich structure allowed ultrasensitive detection of Immunoglobulin‐M with a limit of detection (LOD) of 5 ng/mL due to the enhanced fluorescence of Nile blue by the AuNPs with an optimum distance of 10 nm. Geddes et al. have recently developed a MEF detection systems using different types of 96‐well silvered‐plates.^123^, ^132^ For example, a silvered‐Quanta Plate has been used as a substrate to quantify the amount of trypsin proteolytic enzyme in its solution (i. e., LOD is 1.89 ng). The assay procedure is illustrated in Figure 7b, where a decrease in fluorescence intensity of FITC dye with an increase in the concentration of enzyme was observed. Unlike the typical enzyme‐linked immunosorbent assay (ELISA) method that often require a washing step, this planer MEF immunoassay biosensor can be undertaken without washing due to the MEF effect of silver nanostructures on the 96 well plates.

#### 4.2.2 MEF biosensor designs using colloidal nanoparticles

Developing colloidal MEF biosensors is of great importance since most of the biological phenomena occur in the solution at the nanometer length scale. Like the planar system, developing colloidal MEF systems include three main stages: i) Synthesis of metal core, ii) formation of spacer on metal core with sufficient thickness, and iii) Conjugation of biorecognition element to the as‐formed core/shell nanoparticles. However, stability of the colloidal nanoparticles system is a challenging issue that should be considered in the design of MEF biosensors because undesirable aggregation of metal NPs could alter the MEF response (see *section 4.2.3*).

##### 4.2.2.1 Fluorescent detection of analyte in solution

**Figure 8:**
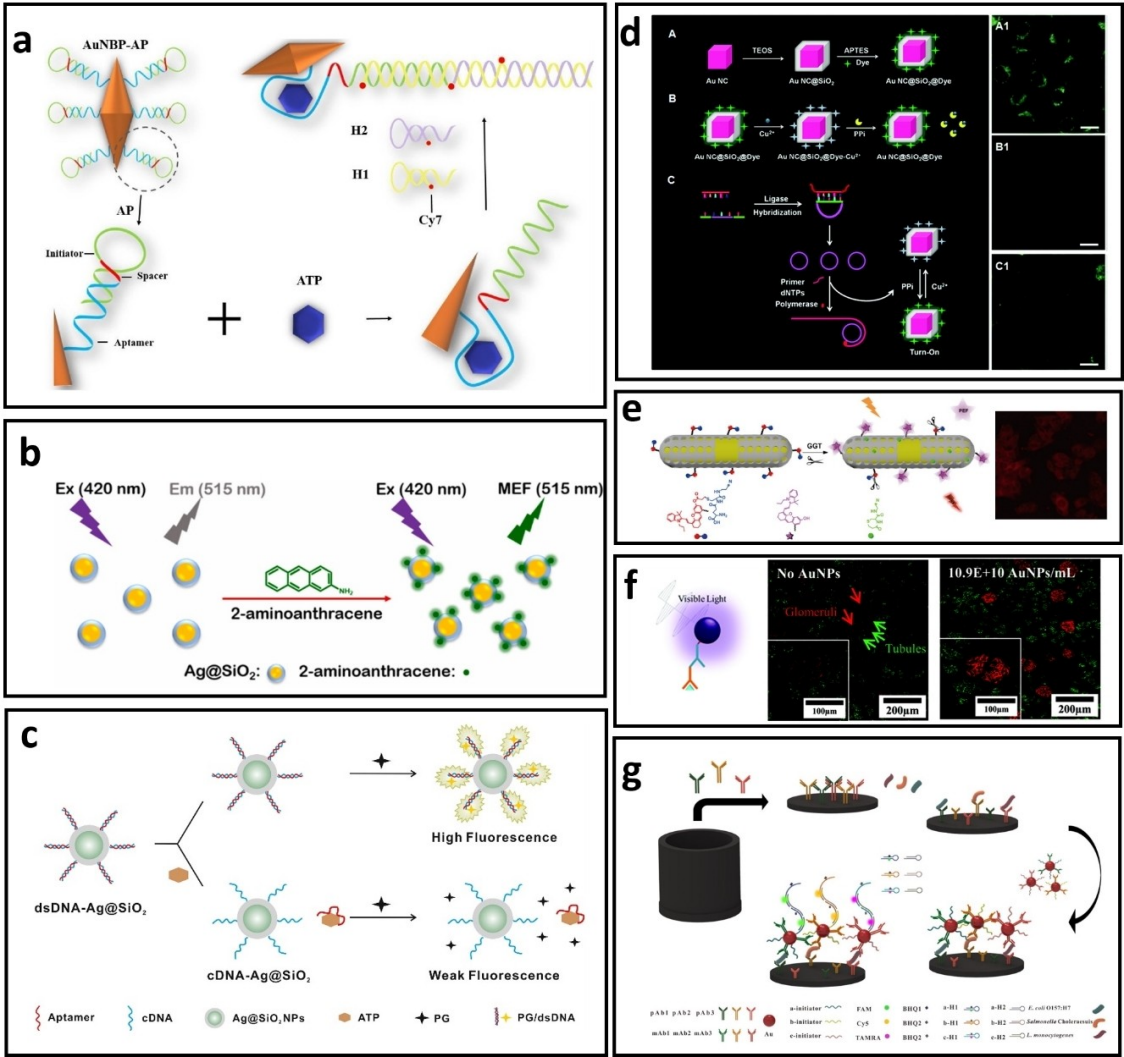
a) DNA‐functionalized gold nanobipyramids (AuNBPs) for the detection of ATP. Interaction between the DNA and ATP results in adjustment of distance between the AuBP and Cy‐7 for fluorescence enhancement,^116^ b) Ag@Silica nanoparticles (Ag@SiO_2_) for metal‐enhanced fluorescence detection of 2‐aminoanthracene,^108^ c) dsDNA‐functionalized Ag@SiO_2_ probes for MEF detection of ATP through a competition assay design. As the ATP molecule interacts with the aptamer leaving the original dsDNA structure, the PicoGreen (PG) dye cannot bind to the cDNA−Ag@SiO_2_, leading to weaker fluorescence,^117^ d) Schematic illustration of (A) the synthesis of Au nanocube@Silica@dye MEF biosensor. (B) Detection of pyrophosphate (PPi). (C) Detection of point mutation based on change in the fluorescence of MEF sensor in the presence of PPi or Cu^2+^,^107^ e) Schematic illustration of the “turn‐on” MEF probe for detecting γ‐glutamyl transpeptidase,^120^ f) Antibody‐functionalized AuNPs as MEF probe for fluorescence imaging. The confocal fluorescence images shows the detection of megalin in the tubules was coloured by the Alexa Fluor 488 (green) while the podocin in the glomeruli was coloured by the Alexa Fluor 647 (red),^127^ g) Schematic illustration of ultrasensitive ELISA based MEF biosensor for simultaneous multicolour detection of pathogens.^121^

Detection and quantification of special molecules existed in water or body‐fluids is essential for analytical applications ranging from diseases diagnostic to monitoring of environmental pollutants. These analytes could be toxic molecules and metal ions in water or radicals, small molecules, and biomarker are related to specific diseases. For example, Zheng et al.^116^ have quantified adenosine triphosphate (ATP) molecules based on its recognition with a specific aptamer linked to the surface of Au nanobipyramid (AuNBP). In this aptamer structure‐switching MEF detection system, APT not only interacts with the aptamer, but also initiates the hybridization chain reaction between the specially designed Cy7‐modified DNA oligonucleotides (H1 and H2), enabling the quantification of ATP molecules with low LOD of 35 nM (Figure 8a).

While the analyte could be different from the fluorophore used in the MEF biosensor, some fluorescent analytes can act as the sensing probe for the ‘turn‐on’ MEF biosensors. For example, Tian et al.^108^ have developed the Ag@SiO_2_ nanoparticles to quantify fluorescent 2‐aminoanthracene molecule which design principle is shown in Figure 8b. As the maximum absorbance of the other interfering molecules (i. e., aromatic amines, some dyes such as methylene blue and Crystal violet) does not overlap with the LSPR peak of the developed Ag@SiO_2_, MEF can occur only in the presence of 2‐aminoanthracene with a detection limit of 1 nM.

The colloidal MEF biosensors can also be designed based on a competition assay. Figure 8c shows the assay principle based on the interaction of ATP molecule with the dsDNA‐functionalized Ag@SiO_2_ nanoparticle and PicoGreen (PG) dye which is a dsDNA intercalator.^117^ The dsDNA probe consists of an aptamer sequence for ATP binding and its complementary DNA (cDNA). In the absence of ATP, the initial fluorescence intensity of the dsDNA‐functionalized Ag@Silica/PG is enhanced due to the MEF effect. As ATP molecule interacts with the aptamer leaving the original dsDNA structure, PicoGreen (PG) dye cannot bind to the cDNA‐conjugated Ag@SiO_2_, leading to a weaker fluorescence. Therefore, the fluorescence intensity of the biosensing probes is inversely proportional to the concentration of ATP in the solution with a wide dynamic detection range (100 nM–10 mM).^117^

##### 4.2.2.2 In‐and in‐vivo fluorescent detection of biomolecules and living organisms

Detection of biomarkers inside the cells is one of the ultimate goals of developing colloidal biosensors for early diseases diagnosis. Cui et al.^107^ have developed Au nanocube@Silica@5‐carboxyfluorescein nanoprobe with a maximum fluorescence enhancement using the 50 nm Au nanocube (core) with 21 nm silica shell thickness. As shown in Figure 8d, the fluorescence of the probe was quenched in the presence of Cu^2+^ ion, while its fluorescence could be recovered upon adding pyrophosphate due to the formation of pyrophosphate–Cu^2+^ complex. As pyrophosphate is a by‐product in the process of rolling circle amplification coupled with the ligase chain reaction, this Au nanocube@Silica@5‐carboxyfluorescein can be employed as the “turn‐on” probe to detect single nucleotide polymorphism (Figure 8d). In another study, Zhang et al.^120^ have developed a dual‐modal biosensor for the detection of γ‐glutamyl transpeptidase (GGT) in living cells. In this design, γ‐glutamyl bond in the molecular probe is cleaved in the presence of GGT, while a self‐immolative fluorescent segment is remained on the surface of Au@silica nanorod, resulting in bright fluorescence due to the MEF effect. The fluorescence intensity is bright enough to visualize the γ‐glutamyl transpeptidase inside the HepG‐2 cells (Figure 8e). Cheng et al.^127^ have developed two sets of plasmonic gold nanoparticles, each functionalized with the anti‐megalin and anti‐podocin for the in‐vivo visualization of megalin and podocin in the tubules and glomeruli. Figure 8f shows the fluorescence images of megalin and podocin in vivo using the two dyes of different colours.

Detection of foodborne pathogens have received tremendous attention because of their serious impacts on human health.^133^ The enzyme‐linked immunosorbent assay (ELISA) is one of the most reliable methods for detecting pathogens. This method relies on the enzymatic reactions and colour changes during these reactions. However, simultaneous analysis of multiple pathogens is difficult due to the weak single colour generated by ELISA method. To address this issue, Lv et al.^121^ have developed an ELISA base on fluorescence hybrid chain reaction, which enables simultaneous analysis of multiple pathogens using dye‐labelled antibodies of different colours. In this MEF biosensor, AuNPs are used to enhance the brightness of dyes to achieve an ultrasensitive detection of multiple pathogens at a lower LOD as compared to the conventional ELISA (Figure 8g).

#### 4.2.3 MEF biosensor design using aggregation‐induced plasmonic hotspots

**Figure 9:**
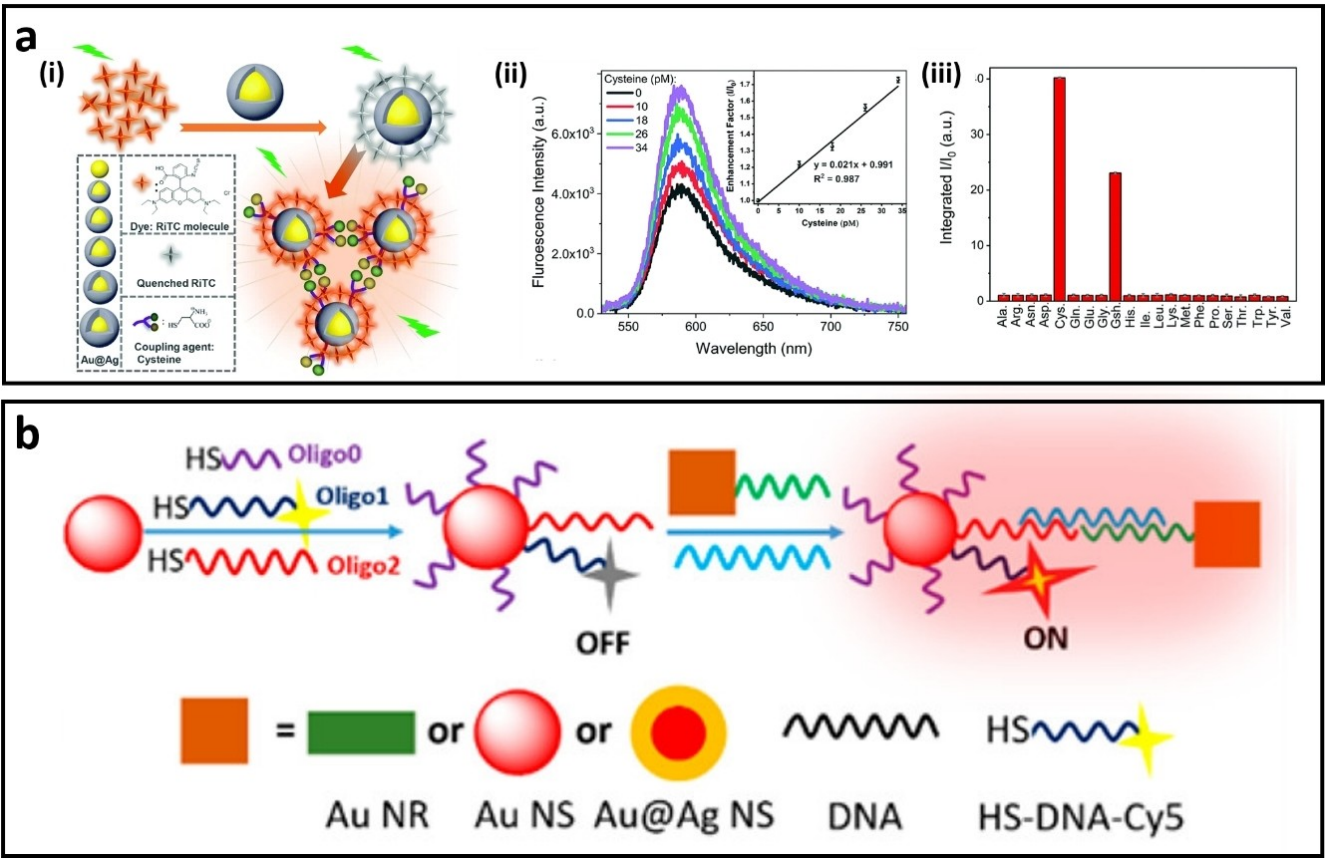
a) (i) Schematic representation of aggregation‐based MEF biosensors using Au@Ag nanoparticles and RITC dye, ii) Fluorescence spectra and enhancement factor (inset) for cysteine detection and iii) Selectivity of cysteine in comparison with other amino acids using the Au@Ag enhanced biosensors.^131^ b) Schematic illustration of the sensing principle based on aggregation‐induced hot‐spots where target DNA can bridge two ssDNA‐functionalized metal nanoparticles and enhance the fluorescence of Cy5 dye molecules in the gap between plasmonic NPs.^130^

Developing MEF‐based biosensors usually requires stability of the colloidal system in biological media. However, aggregation of metal NPs is not always an unfavourable process. Aggregation of metal NPs could result in the formation of hot‐spots, which in turn enhancing the excitation rate in the fluorophore with stronger enhancement. The aggregation of metal NPs can be induced through physical or chemical interactions triggered mostly by the target analytes. For example, cysteine molecules have been used to induce the aggregation of dye‐functionalized Au@Ag nanoparticles, resulting in the formation of hot‐spots between the bimetallic nanoparticles (Figure 9a**)**. While the fluorescence of rhodamine B isothiocyanate (RITC) is quenched on the surface of individual Au@Ag nanoparticles, its fluorescence is “turned on” after forming plasmonic hot‐spots, allowing determination of the concentration of cysteine molecules in the colloidal MEF biosensor (Figure 9b & c).^131^ Another method is to bridge the metal NPs using oligonucleotides. In this approach, different types of metal NPs such as gold nanorods (AuNR) or nanosphere (AuNS) are gathered together to form the plasmonic hotspots. Figure 9d illustrates the sensing principle of this MEF biosensors for DNA detection through aggregation‐induced plasmonic hotspots. Specifically, the fluorescence intensity of Cy5‐T10‐(CH2)3‐SH DNA is quenched when conjugated onto the AuNS. However, its fluorescence was “turned on” when the target DNA with complementary sequence bridging the Cy5‐T10‐(CH2)3‐SH DNA‐functionalized AuNS to another ssDNA‐modified AuNPs (i. e., AuNR, Au NS, and Au@Ag NS) to form the hotspot between the gap area of two metal NPs. The results suggested that bimetallic NPs hotspot could enhance fluorescence more effectively than the AuNR or AuNS alone in the MEF biosensing system.^130^

## 5 Biosensor designs based on surface‐enhanced Raman scattering (SERS)

### 5.1 Principles of SERS

When a molecule is irradiated by a high intensity laser light source, most of the scattered light is at the same wavelength of the incident light. Only a small portion of the scattered light (typically 0.0000001%) has a wavelength different from the laser wavelength. This provides useful information about chemical structure, phase and polymorphy, crystallinity and molecular interactions of the studied molecule. This non‐destructive chemical analysis technique is called Raman spectroscopy. However, the Raman spectrum is usually weak at low concentrations of analyte, which restricts the use of Raman spectroscopy for biosensing applications when the analytes are usually low in quantities.

Surface‐enhanced Raman scattering (SERS) is a physical phenomenon when the Raman scattering signal of a molecule is enhanced by the plasmonic nanoparticles. The typical enhancement factor for SERS is about 10^4^–10^8^‐fold, which is highly sensitive to allow detection of biological analytes at ultralow concentration and even at the single molecular level. There are electromagnetic field and chemical enhancement mechanisms in SERS. The electromagnetic field enhancement refers to the enhanced excitation rate in the Raman probe as a result of the enhanced electric field around the metal nanostructure under the excitation laser. It has been reported that the excitation rate is proportional to the power of four of the enhanced electric field ($\frac{{\left|E\right|}^{4}}{{\left|E_{0}\right|}^{4}}$ ) at the position of Raman probe. This factor enables the researchers to predict the best size, shape and even the percentage of surface coverage on metal NPs for the maximum SERS enhancement factor via computational simulations.5b, 134 The chemical mechanism refers to the Raman enhancement associated with the electron or charge transfer process between the ground/excited state of Raman probe and metal nanoparticle.^135^ The selection of Raman laser wavelength also depends on the nature of Raman probe and its optical properties (i. e., absorption cross‐section and fluorescence), as well as the sensitivity of the molecule to the excitation wavelength.^136^

### 5.2 Types of SERS biosensors

**Figure 10:**
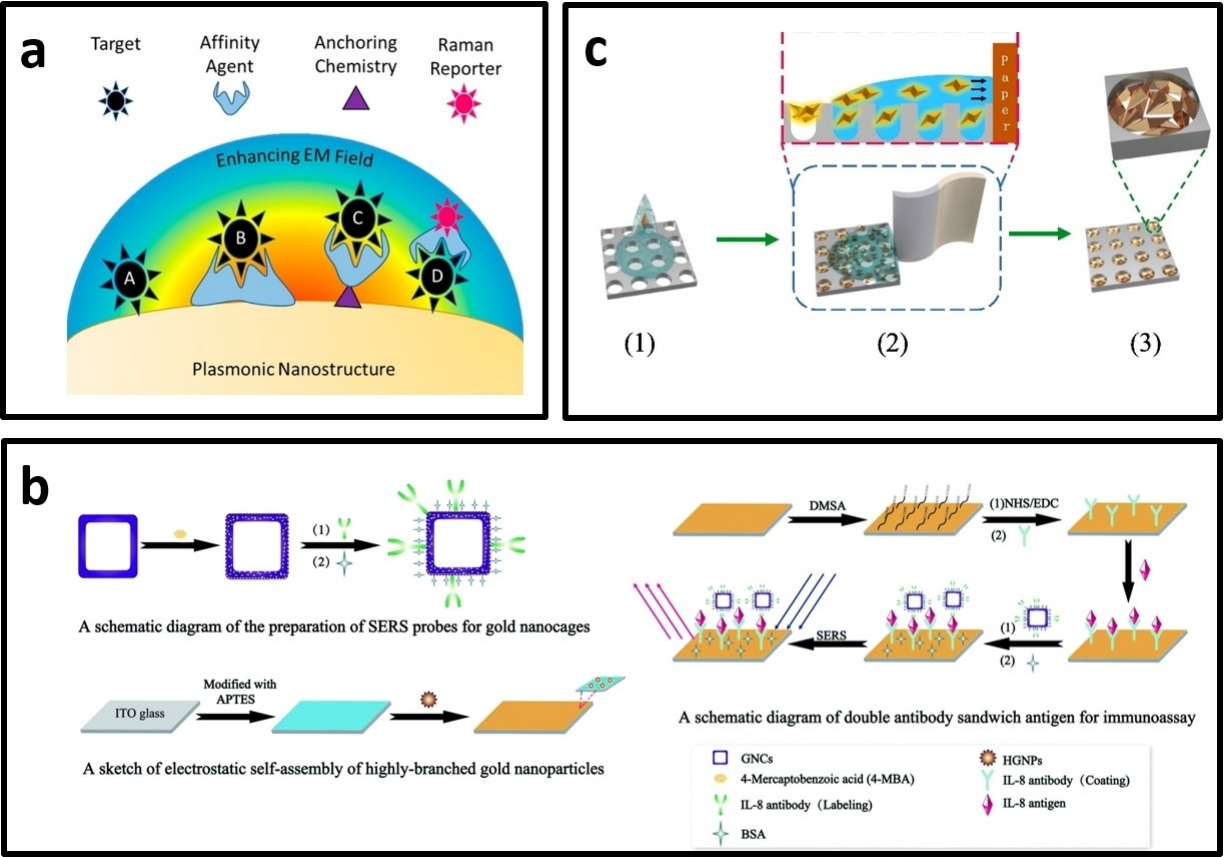
a) Different approaches for the development of selective SERS probes F,^93^ b) Developing planar SERS substrate using antibody‐functionalized Au nanocages (in the solution) and Au nanostars (self‐assembled on the surface of ITO glass). The sandwich structure enhances the Raman signal from Raman reporter molecules on the surface of Au nanocages and Au nanostars,^171^ c) Fabrication of planar SERS substrate by the deposition of Au bipyramid (AuBPs) inside the nanoholes of anodic aluminium oxide for detection of detect the aflatoxin B1.^153^

Figure 10a shows the different approaches for the development of selective SERS probes. Some of the recent advances in the SERS biosensors have been summarized in Table 2. As can be seen, anisotropic nanoparticles have extensively been used in developing SERS biosensors for various bioanalytes ranging from small molecules, RNA/DNA to proteins and even living cells, showing its potential for biomedical diagnostics where the biomarkers are often very low in concentration.

**Table 2:** Recent advances on developing SERS biosensors using gold and silver nanostructures.

Nanoparticle	Colloidal or Planar	Raman Reporter	Excitation wavelength	Analyte	Linear Range	LOD	Ref
Hexoctahedral Au @AgPt nanoparticles/300 nm×90 nm	Colloidal	6‐Mercaptohexanol	785 nm	Hg^2+^ ion	1 nM–10 μM	0.28 nM	137
Ag‐TiO2 Nanoparticle/6–8 nm	Colloidal	Difloxacin hydrochloride	633 nm	Difloxacin hydrochloride	1×10^−4–^1×10^−11^ M	4.36 pM	138
Ag nanoparticle/55 nm	Colloidal	polycyclic aromatic hydrocarbons	785 nm	polycyclic aromatic hydrocarbons	50–250 ng/L	0.1–100 μg/L	139
ZnO nanorod@Ag nanoparticles ZnO NR: 7.5 μm×800 nm Ag NP: 58 nm	Planar	Rhodamine 6 G	785 nm	pioglitazone phenformin	10–3 to 5×10–9 M 10–3 to 10–8 M	1 nM 5 nM	140
Fe3O4/Au@ATP@Ag Nanorod sandwich structure AuNR: 85 nm×23 nm Ag thickness: 4.2 to 13.5 nm	Colloidal	4‐Aminothiophenol (ATP)	–	Histamine	10^−8^–10^−3^ M	1×10^−8^ M	141
Au@Ag nanorod/49 nm×16 nm	Planar	4‐aminobenzothiol	633 nm	Thiram	–	1×10^−15^ M	142
ZnO nanorod@Au NP ZnO NR: 70 nm diameter Au NP: 10 nm	Planar	methyl blue crystal violet	633 nm	methyl blue crystal violet	10^−9^–10^−4^ M 10^−7^–10^−11^ M	1 nM 0.01 nM	143
Au nanorod/75 nm×17 nm	Colloidal	Cy3	785 nm	Pb^2+^ ion	0.5–100 nM	0.01 nM	144
Au nanorod/23 nm×6 nm	Planar	Cocaine	780 nm	Cocaine in oral fluid sample	–	10 ng/mL	145
Au nanorod	Planar	Cysteamine		acephate			146
Au nanorod/106 nm×42 nm	Planar	thiabendazole	785 nm	thiabendazole	0.1–100 ppm	0.037 mg/L	147
Au nanorod Length: 46.7 nm Width: 13.3 nm	Planar	methylene blue malachite green	633 nm	methylene blue malachite green	0.5 and 0.1 ng/mL	0.5 ng/mL 0.1 ng/mL	148
Ag nanoplate/80 nm	Planar	Rhodamine B	785 nm	H_2_S	82–330 nM	‐	149
Ag nanoplate/20–80 nm	Planar	thiram	532 nm	thiram	0–7.5 mgkg^−1^	0.7 ppm	150
Ag nanoplate/30–60 nm	Planar	Thiram methyl parathion	514 nm (effect of excitation)	Thiram methyl parathion	106 nM–10 nM 5×105 nM–1×103 nM	40 nM 600 nM	151
Au bipyramid/120 nm×50 nm	Planar (Tape)	methyl parathion	785 nm	methyl parathion	–	31.56 ng/cm2	152
Au bipyramid/70 nm×22 nm	Planar (AAO)	Aflatoxin B1	785 nm	Aflatoxin B1	1.5 μg/L to 1.5 mg/L	0.5 μg/L	153
Au@Ag hollow nanocubes/120 nm	Planar (ITO)	uric acid (UA) ascorbic acid (AA)	532 nm	uric acid (UA) ascorbic acid (AA)	0.05–2 mM 0.05–0.5 mM	0.36 μM 0.019 μM	154
Ag nanocube	Planar (PDA)	Deoxynivalenol	633 nm	Deoxynivalenol	1 fM to 1 mM	0.82 fM	155
Ag@SiO2 nanocubes	Planar	Aspartame	633 nm	Aspartame	0.2–1 mgmL^−1^	71 μgmL^−1^	156
Gold‐silver bimetallic nanotrepangs	Colloidal	2‐mercaptopyridine 4‐aminothiophenol 4‐nitrothiophenol	785 nm	Exosome in different cell lines	–	26 Particles/μL	157
Au@AgAg bimetallic Nanorods	Colloidal	2‐mercaptopyridine	785 nm	Oligonucleotides (HPV‐16)	1 fM–1 pM	1 fM	158
Au@Ag nanocuboids/Au core: 23 nm, Ag shell: 78 nm	Planar	Butyl benzyl phthalate Diethylhexyl phthalate	633 nm	Butyl benzyl phthalate Diethylhexyl phthalate	–	1 nM 1 nM	159
Ag bumpy nanoshell@Silica	Colloidal	Mixture of 4‐fluorobenzenethiol (4‐FBT), 4‐bromobenzenethiol (4‐BBT) and 4‐chlorobenzene‐thiol	785 nm	lymph nodes (In‐Vivo)	–	–	160
AuNS@Ag@SiO2 Nanostars	Planar	4‐mercaptobenzoic acid	633 nm	a‐fetoprotein	3 pg mL^−1^ to 3 mg mL^−1^.	0.72 pg/mL	161
Ag nanocube/50 nm	Planar	4‐mercaptophenyl boronic acid	785 nm	Dopamine	10^−13^–10^−4^ M	40 fM	162
(Au nanorod@Ag)‐polyaniline Janus Nanoparticles/60–70 nm length and 30–40 nm width	Colloidal	polyaniline	532 nm	Hg^2+^ ions	1–150 nM	0.97 nM	163
Concave AuAg nanowalls/60 nm	Colloidal	4‐Nitrothiophenol	785 nm	malachite green	50 fM–100 pM	50 fM	164
Au@Ag nanocube/67 nm	Planar (PDMS)	Imazalil	785 nm	Imazalil	–	1 ppm	165
Au nanostar@4‐MBA@Au nanoshell/50 nm	Colloidal	4‐mercaptobenzoic acid (4‐MBA)	633 nm	Exosome	40–4×107 particles/μL	27 particles/μL	166
Au nanostar@Raman Reported@Silica/25 nm Au core with 2–3 nm silica shell	Colloidal	4‐nitrothiophenol Diamino‐1,3,5‐ triazine‐2‐thiol	785 nm	MDA‐MB‐231 and MCF‐7 breast cancer cells	–	–	167
Au nanostar/70 nm	Colloidal	Indocyanine green	785 nm	Mapping of U87 glioma cells	–	–	168
Au@Ag nanostar‐Au nanopartciles Core‐Satellite/68 nm Au@Ag NS and 13 AuNP	Colloidal	4‐mercaptobenzoic acid	633 nm	adenosine triphosphate	1 pM–1 nM	0.5 pM	169
Au nanostar/138 nm	Colloidal	Cysteine residue in the protein	633 nm	avb3 integrin on human metastatic colon cancer cells	–	–	170
Au Nanocages/Au nanostar Sandwich Au nanocage: 30 nm Au nanostar: 600 nm	Planar	4‐mercaptobenzoic acid	785 nm	Interleukin 8 (IL‐8) gene	10 pg/mL–1 mg/mL	6.88 pg mL^−1^	171
Au nanostar/60–70 nm	Planar	1,2‐bisĲ4‐pyridyl)ethylene (BPE) and 4‐mercaptobenzoic acid	785 nm	Zika Virus Dengue Virus	–	–	172
Hollow hydrangea Au Nanoparticles/Tunable size (170 nm–550 nm)	Coloidal	4‐nitrothiophenol	633 nm	4‐Nitrothiophenol (monitoring reaction)	–	–	173
AgNWs−Au NPs core‐satellite AgNW: 60 nm diameter, Au NPs: 5 nm	Planar	293T‐Mig‐R1 293T‐Mig‐2 C9 cells	785 nm	293T‐Mig‐R1 293T‐Mig‐2C9 cells	–	–	174
AgNW@AgNP AgNW: diameter	Planar (on site : fish and leaf)	malachite green thiram	633 nm	malachite green thiram	–	0.01 nM 0.1 nM	175
Au nanowire on Au film/200 nm diameter	Planar	Cy5	633 nm	miR141 and miR375 from prostate cancer cells	100 aM–100 pM	100 aM	176
Au nanorood/ length of 60 nm and 33 nm	Planar	Thiram	785 nm	Thiram	10–4 to 10–7 M	1.2 nM	177
Au nanorod@Silica / Length: 49 nm Diameter: 18 nm	Colloidal	A designed organic Raman Reporter	785 nm	γ‐Glutamyl Transpeptidase in cells	0–60 U/mL	1.2×10–3 U/L	120
Au nanobone/80 nm×27 nm	Colloidal	rhodamine B	633 nm	Escherichia coli O157:H7	10–10,000 CFU/mL	3 CFU/mL	178
AuAg nanostar	Colloidal	DNAs	785 nm	DNA Mutation Detection	–	1 input copy	179
Au@Ag nanorod	Planar	4‐MBA	532 nm	Total Prostate specific antigens	2–120 fg/mL	0.94 fg/mL	180
Au nanorod/50 nm in length and 13 nm in width	Colloidal	a new kind of bioorthogonal Raman reporter	785 nm	MCF‐7 cells	–	–	181
Au nanorod/55 nm in length and 18 nm in diameter	Colloidal	MO R6G FITC	785 nm	MO R6G FITC	0–10 μM	–	182
Au NR Dimers/70 nm×50 nm	Colloidal	Gliadin biotinylated‐IgG Antibody Ara h1 biotinylated‐IgG Antibody	633 nm	Gliadin biotinylated‐IgG Antibody Ara h1 biotinylated‐IgG Antibody	–	pM Range	183
Waxberry Au Core‐AgNP satellite/140 to 170 nm	Colloidal	β‐mercaptoethylamine	780 nm	thiram (in vivo)	10–7 to 10–4 M	–	184
Ag@Fe3O4 NPs/253.3 nm with 80.9 nm AgNP core	Colloidal	malachite green isothiocyanate 7‐dimethylamino‐ 4‐methylcoumarin‐3‐isothiocyanate 4‐(1H‐pyrazol‐4‐yl)‐pyridine	532 nm	Escherichia Coli Salmonella typhimurium methicillin‐resistant Staphylococcus aureus	10‐10^7^ CFU/mL	10 CFU/mL	185
Au nanorod/45 nm×12 nm	Planar	R6G E. Coli	532 nm 785 nm	R6G E. Coli	10^−11^–10^−9^ M	<10 pM	186
Au Bipyramids/117.05 in length and 36.08 in width	Colloidal	2‐naphthalenethiol	785 nm	MCF‐7 cell	5–500 cells/mL	5 cells/mL	187
							
Au Nanoplate‐Au nanoparticle Sandwich/20–30 μm of edge length and 100–200 nm of thickness	Planar	Cys3	633 nm	C‐reactive protein	–	10–17 M	188
Ag nanoplate/12.5 nm edge length	Planar	Thiram	633 nm	Thiram in soil	0.12 to 4.8 μg/g	90 ng/g	189
Au@Ag Nanoparticles/30 nm core, 5 nm Ag thickness	planar	Rhodamine 6G E. coli P. aeruginosa S. aureus	633 nm	Rhodamine 6G E. coli P. aeruginosa S. aureus	10^−9^–10^−3^ M – – –	1 nM – – –	190
AgAu nanocage/43 nm edge length	Planar	4‐mercaptophenylboronic acid	633 nm	CEM Cell microRNA‐21	10–10000 cells 10–12–10–8 M	1 cell 166 fM	191
Ag@Au nanoparticle/15–20 nm	Coloidal	4‐aminothiophenol 4‐Nitrothiophenol	785 nm	ochratoxin A aflatoxin B1	0.05–100 ng/mL 0.05–100 ng/mL	0.006 ng/mL 0.03 ng/mL	192

#### 5.2.1 Planar SERS biosensors

In this review, we only focus on design of planar SERS biosensors based on the binding of colloidal nanoparticles onto the surface of substrate due to its simplicity and potential of large‐scale fabrication. The glass and silicon substrates are popular for developing planar SERS biosensors. The number of hotspots could be controlled by the number of metal NPs bound on the surface of substrate. Hamad‐Schifferli's group has immobilized the Zika and Dengue antibodies‐functionalized Au nanostars and Raman reporter on the surface of glass to interact with the viruses.^172^ Wang et al.^171^ have developed a planar SERS system for detecting Interleukin 8 (IL‐8) in human serum by depositing the Au nanostars on ITO glass, followed by functionalization with the IL‐8 antibody. The (IL‐8 antibody and Raman reporter)‐co‐functionalized Au nanocages was then used to detect the IL‐8 molecules on the SERS substrate. This sandwich structure resulted in the enhanced electric field between Au nanocage and Au nanostar for sensitive detection of IL‐8 molecules (Figure 10b). Using a similar approach, Su et al.^191^ have developed a ratiometric SERS biosensor for the detection of CEM cells and microRNA‐21. In this design, H_2_O_2_ induces deboronative hydroxylation of the Raman reporter (i. e., 4 mercaptophenylboronic acid), leading to a stable change in the SERS intensity between the two main peaks at 998 cm^−1^ and 1074 cm^−1^ of this molecule.

Recently, planar SERS sensors using other substrates such as silicon (Si) wafer have been reported. Lin et al.^153^ have used the anodic aluminium oxide (AAO) as a template to form the Au bipyramids (AuBPs) hotspots. They dropped the solution of AuBPs into the nanoholes of AAO and dried the solvent by a paper, causing the aggregation of AuBPs into the nanoholes and formation of hot‐spots between them (Figure 10c). The as‐developed SERS substrate was then used to detect the aflatoxin B1 in the peanut extract with a LOD of 0.5 μg/L. Zhao et al.^165^ reported the use of polydimethyl soloxane (PDMS) to fabricate a flexible SERS substrate. The Au@Ag nanocubes were deposited on the surface of Si wafer, followed by covering with a layer of PDMS. The PDMS film was then peeled off for the in‐situ detection of imazalil (LOD=1 ppm).

#### 5.2.2 Colloidal SERS biosensors

The colloidal SERS biosensors are ultrasensitive diagnostic tools that could be used for the detection and quantification of a wide range of analytes from ions and small molecules to bio‐macromolecules, viruses and pathogenic bacteria. Depending on the design of biosensor, the analyte could directly interact with the functionalized metal nanoparticle where the Raman spectra of the analyte can be measured and used for the quantification of the concentration of analyte in the sample. However, incorporation of a Raman reported molecule into the design (i. e., either on the surface of metal nanoparticle or inside the shell around the nanoparticle) is necessary to correlate the existence of the analyte molecule to the Raman spectra of Raman reporter.

##### 5.2.2.1 SERS detection of analyte in solution

**Figure 11:**
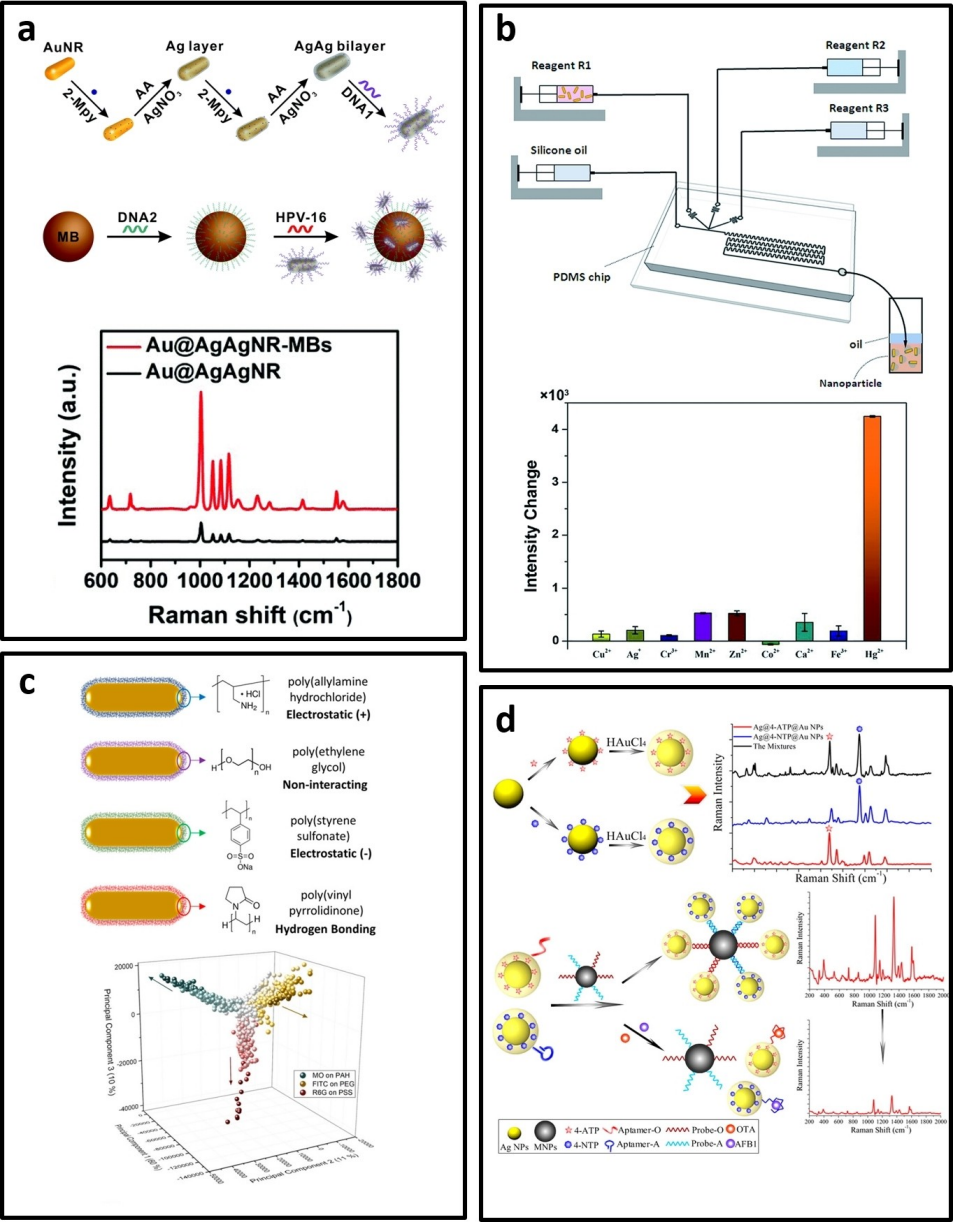
a) Schematic illustration of colloidal biosensor using DNA‐functionalized Au@AgAg nanorod as SERS probe (top) for the detection of gene HPV‐16 using magnetic bead (bottom),^158^ b) Synthesis of anisotropic Au nanorod/Polyaniline nanoparticles by a microfluidics approach (top) and change in the Raman intensity in the presence of different ions (bottom),^163^ c) development of polymer‐coated Au nanorod for simultaneous detection of different Raman active analytes (MO, FITC and R6G) via electrostatic interaction or hydrogen binding,^182^ d) Simultaneous detection of ochratoxin A and aflatoxin B1 by self‐assembly of Au nanoparticles around magnetic nanoparticle via the hybridization of designed aptamers and their complementary DNAs.^192^

Colloidal SERS biosensors have extensively been used for the detection of different analytes in the solution. The sample solution containing the analyte can be an extract from a real sample (i. e., mostly for food and agricultural applications) or a body‐fluid from human or animal (i. e., for biomedical applications). Ning et al.^158^ functionalized the Au@Ag core‐shell nanorods with a Raman reporter molecule and specific DNA (i. e., DNA1) to detect the HPV‐16 gene. In parallel, a magnetic bead has also been functionalized with another DNA (i. e., DNA2), where the two designed DNAs were complementary to the target gene of HPV‐16. The addition of gene HPV‐16 and the DNA1‐functionalized Au@AuAg nanorods to the DNA2‐functionalized magnetic bead in solution resulted in the binding of nanorods on the surface of magnetic bead. This binding not only enhances the electric field between the nanorods, which is favourable for SERS detection, but also enables the separation of the gene HPV‐16 from the solution (Figure 11a). Similar approach has been applied to capture and detect the exosome from human serum using gold nanostar as SERS enhancer and magnetic beads for capturing as well as aggregating the Au nanostars.^166^ In another example, Wang et al.^163^ have developed an Au@Ag nanorod‐polyaniline Janus nanoparticle via a microfluidics approach for the SERS detection of Hg^2+^ ions in aqueous solution. The strong coordination between the nitrogen atom of polyaniline and Hg^2+^ ions resulted in a lower stability of the designed Janus nanoparticle, leading to particle aggregation. The formation of hot‐spots between the Au@Ag nanorods as a result of aggregation enhances the Raman spectra of polyaniline, which was proportional to the concentration of Hg^2+^ ions (Figure 11b).

On the other hand, metal NPs functionalized with different recognition elements allows the simultaneous detection of multiple analytes, which approach is also known as the plasmonic nose. However, the analytes or the Raman reporters used in this approach should have a distinct Raman peak to better differentiate the molecules by SERS spectra. As shown in Figure 11c, Yilmaz et al.^182^ have functionalized the Au nanorods with three different polymers including the positively charged poly(allylamine hydrochloride) (PAH), negatively charged poly(styrenesulfonate) (PSS), and poly (vinylpyrrolidone) (PVP). The PAH‐AuNRs and PSS‐AuNRs could interact with the methyl orange (R6G) through electrostatic interactions, while the PVP‐AuNRs could interact with FITC dye via hydrogen bonding. Therefore, the mixture of these three types of functional AuNRs can be used to detect the three dyes in their ternary mixture In addition, the use of different Raman reporters can enable simultaneous detection of two mycotoxins, i. e., ochratoxin A and aflatoxin B1 (Figure 11d).^192^ Au@Ag nanoparticles were functionalized with a pair of 4‐aminothiophenol and ochratoxin A aptamer or 4‐Nitrothiophenol and aflatoxin B1 aptamer. A magnetic bead functionalized by the complimentary aptamer sequence resulted in the aggregation of Au@Ag NPs with enhanced Raman signals. The application of colloidal SERS biosensors is not only limited to the detection of trace amount of an analyte, but also allowed the monitoring of the reaction for the quantifying the analyte concentration within the reaction period. For example, Qin et al.^173^ have used the hollow hydrangea AuNPs to monitor the catalytic reduction of 4‐nitrothiophenol to 4‐aminothiophenol.

##### 5.2.2.2 In‐vivo SERS detection and imaging of living cells

**Figure 12:**
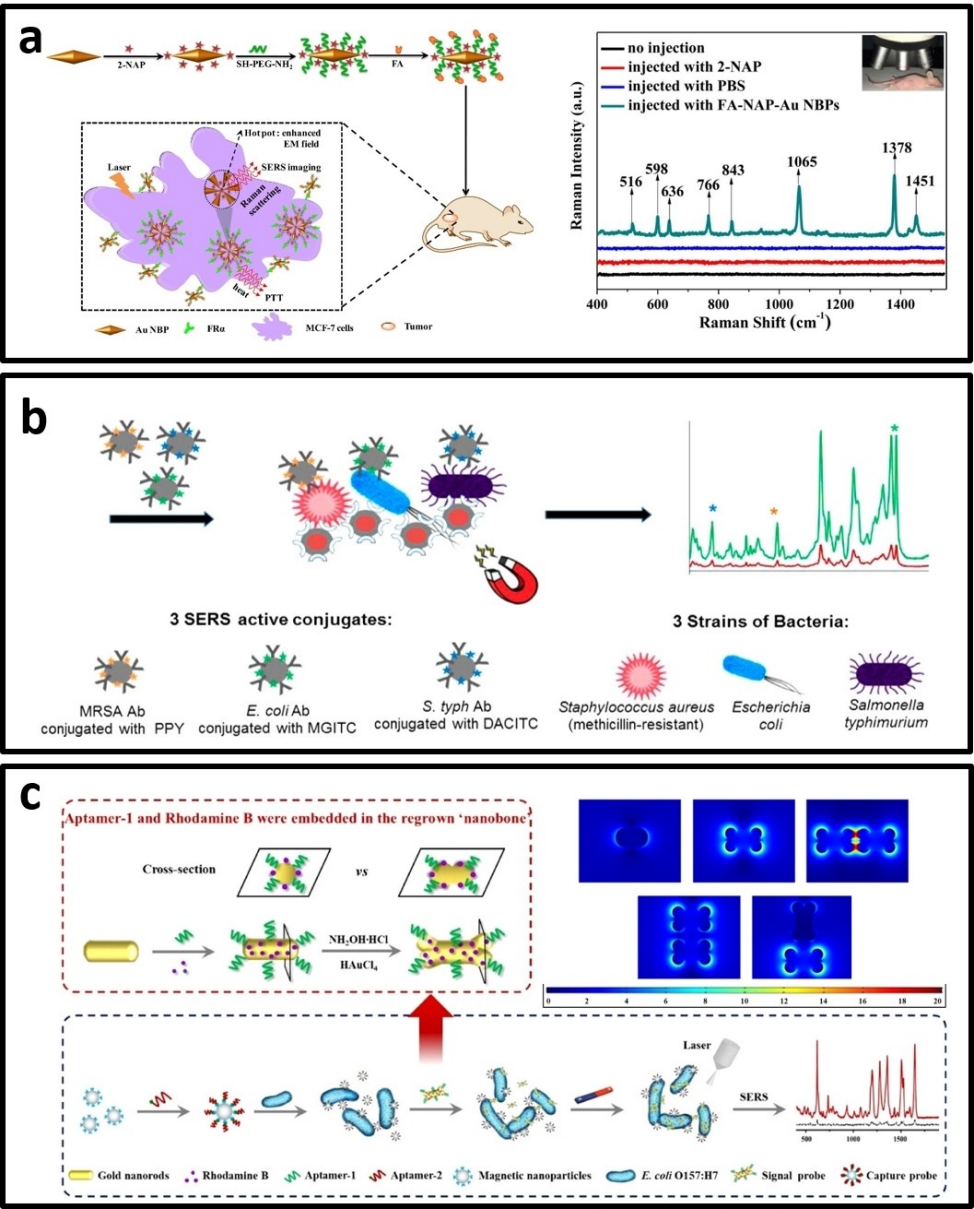
a) Schematic illustration of the preparation folic acid‐functionalized gold bipyramid nanoparticles (AuNBPs) loaded by Raman reporter and its application in SERS detection of MCF‐7 breast cancer cells,^187^ b) Antibody‐functionalized Ag@Fe_3_O_4_ nanoparticles for multiplex detection of different strains of bacteria,^185^ c) Au nanobone functionalized with aptamer and Raman reporter for the detection of *E.coli* pathogen. The top right images shows the enhanced electric field around the Au nanobone in single and dimer configurations.^178^

Colloidal SERS biosensors are especially suitable to detect the biomarkers for in‐vivo diseases diagnosis, monitoring of specific biochemical phenomenon inside the cells as well as detecting live pathogenic bacteria. For example, gold nanostars have been used to study the interaction between avb3 integrin on human metastatic colon cancer cells and the arginine−glycine−aspartic acid (RGD) peptide. The Raman signal from the cysteine residue in the protein allows to monitor this integrin‐specific interaction via SERS.^170^ In another study, Zhang et al. have designed a specific organic molecule attached to the surface of Au@silica nanorod with enhanced Raman signal correlated to the amount of γ‐glutamyl transpeptidase inside the cell.^120^ The SERS probes can also be used for the visualization of cancer cells. For example, Feng et al.^187^ have synthesized the 2‐naphthalenethiol and folic acid co‐functionalized Au bipyramids for simultaneous SERS imaging and photothermal therapy of MCF‐7 breast cancer cells. As folic acid allows specific interaction with the folate receptor alpha on MCF‐7 cell, the folic‐acid functionalized SERS probe has been employed for in‐vivo detection of tumour inside the mouse with a strong Raman signal (Figure 12a).

Detection of pathogens is of interest and importance in terms of food safety and public health. Despite the progress in developing planar SERS biosensors for the detection of multiple pathogens, many of them require complex separation steps well as dropping of sample on the planar substrate, which is cumbersome and time‐consuming.^193^ The colloidal SERS system has the unique features to address these drawbacks and reduce the time of analysis. For instance, Kearns et al.^185^ have developed a colloidal sandwich structure using Ag@Fe_3_O_4_ nanoparticles to detect three different pathogens (i. e., Escherichia Coli, Salmonella typhimurium, and methicillin‐resistant Staphylococcus aureus). In this biosensor design, Ag@Fe_3_O_4_ NPs were functionalized with a pair of antibody and Raman reporter specific for each pathogen separately. The mixture of functionalized nanoparticles was then used to detect three kinds of pathogens simultaneously, achieving a wide range of linear response (10–10^7^ CFU/mL) and low limit of detection (10 CFU/mL) (Figure 12b). To further decrease the limit of detection, Zhou et al.^178^ have applied the similar approach by replacing Ag@Fe_3_O_4_ with a unique anisotropic gold nanostructure (i. e., Au nanobones) as the plasmon enhancer for SERS. The enhanced electric field of Au nanobones led to ultrasensitive SERS detection of Escherichia coli O157 : H7 with a LOD of 3 CFU/mL (Figure 12c).

## 6 Conclusion and perspective

Synthesis of anisotropic nanoparticles, especially gold and silver, has witnessed a huge progress within the last twenty years. Different methods have been developed to synthesize highly monodispersed nanoparticles with good control of morphology. As the optical properties of metal nanoparticles largely depend on their size and shape, it is important to develop reliable synthesis approach to produce a variety of metal NPs in high yield suitable for different applications. In particular, anisotropic metal NPs possess dramatic enhanced electric field at their sharp corners and edges under a wide range of excitation wavelengths, from visible to near‐infrared region, making them highly suitable to be used as plasmon enhancer in metal‐enhanced fluorescence (MEF) and surface‐enhanced Raman scattering (SERS). Among the optical biosensors, MEF and SERS stand out to be the most sensitive techniques to detect wider range of analytes from ions, biomolecules to macromolecules and even living microorganism. Besides the robust synthesis of anisotropic nanoparticles for plasmon enhancement, design of metal‐enhanced biosensors also depends on the selection or screening of specific biorecognition elements such as aptamers or antibodies for targeted bio‐detection. The right combinations of metal nanoparticles, biorecognition element as well as their functionalization and assembly will lead to successful designs of MEF and SERS biosensors.

The applications of SERS and MEF biosensors have increased rapidly during last few years. However, the need for high‐tech instrumentations as well as highly diluted samples have hindered their progress toward clinical diagnostics. Some of these limitations could be addressed by developing brighter MEF and stronger SERS probes as well as different assay designs including the aggregation‐induced plasmonic hotspots formation through bio‐directed or self‐assembly. In addition, microfluidic and capillary (i. e., paper‐based) systems can be used to develop high quality metal nanoparticle for biosensor development with high reproducibility and reliability in detection. Despite the successful development of anisotropic nanoparticles of various shapes, limited number of these nanoparticles have been used in MEF and SERS biosensors. This shows a research gap to be addressed in the future, especially the development of next generation metal‐enhanced biosensors which require high selectivity, ultrasensitivity and great accuracy in the diagnostic results.

For in‐vivo applications towards clinical diagnosis, MEF and SERS nanoprobes also restricted by the relevant issues such as strong background signals from the biological tissues as well as the penetration depth of laser beam to reach the targeted site (e. g., tumor) in living body. To address these issues, different groups of researchers have been working on the development of 1) new nanomaterials with suitable absorbance and emission in biological windows to minimize these background signals and 2) new generation of laser beams to have a deeper penetration in biological tissues. Besides Raman signal or fluorescence enhancement, metal nanostructures can also enhance other properties such as singlet oxygen generation (SOG) by placing the photosensitizer near to the metal NPs. This opens the door for the development of multifunctional nanomaterials with both diagnostic (e. g., MEF bioimaging) and therapeutic functions (e. g., ME‐SOG) for image‐guided photodynamic therapy. Moreover, anisotropic gold nanostructures such as Au triangular nanoplates with flat surface (for better adsorption on tumors/cancer cells) and absorption in the NIR region can also be employed as the photothermal therapy agent *cum* plasmon‐enhanced nanosensing probes for theranostic treatment.

We believe that the metal‐enhanced biosensors including MEF and SERS systems as reviewed herein possess promising potentials to be the new generation theranostic tools in nanomedicine. Its success for practical applications strongly depends on the accomplishment in the development of new theories, modelling and simulation of these processes as well as standardizing different procedures for the fabrication of substrates (i. e., both planar and colloidal systems) and data analysis in the real and complex mediums, and finally, systematic studies for clinical translation and human testing.

## Conflict of interest

The authors declare no conflict of interest.

## Biographical Information

*Yen Nee Tan is an Associate Professor of Chemical Engineering at the Newcastle University. She obtained her PhD degrees from MIT and National University of Singapore under Singapore‐MIT Alliance Scholarship. She is also the Principal Investigator of Biosensors and Nanomaterials at the Newcastle Research & Innovation Institute (NewRIIS) in Singapore. She has authored over 50 journal publications, book/chapters and hold 18 patents on nanobiotechnologies for analytical and biomedical applications. Her current research focuses on the development of multifunctional bio‐hybrid nanomaterials and green technologies inspired by Nature for biomedicine, environmental detection and food security purposes*.

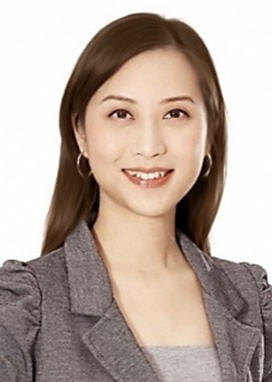

## Biographical Information

*Mohammad Tavakkoli Yaraki received his Ph.D. in chemical and biomolecular engineering from National University of Singapore under the supervision of Prof Yen Nee Tan and Prof Bin Liu. His Ph.D. thesis was about the synthesis of metal nanoparticles and applications for metal‐enhanced singlet oxygen generation of photosensitizers with aggregation‐induced emission. The outcomes of his research have been published in several peer‐reviewed journals. His current research interest is design and development of hybrid nanomaterials containing plasmonic metal nanoparticles for various analytical purposes as well as biomedical applications*.

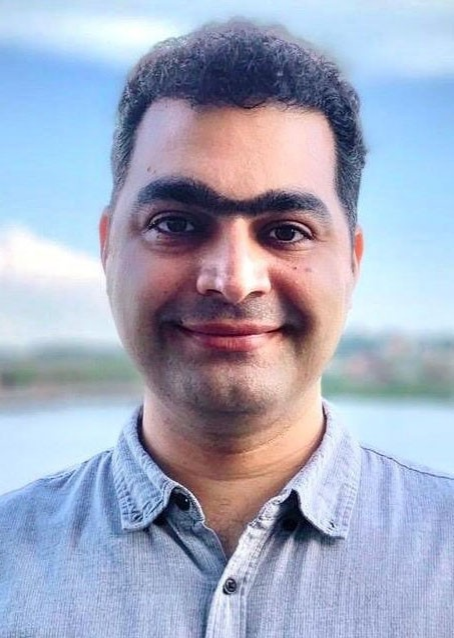
